# Shenqi Granules Ameliorate Cerebral Ischemia-Reperfusion Injury by Regulating Glucocerebrosidase and Uridine Metabolism in Microglia

**DOI:** 10.3390/metabo16100802

**Published:** 2026-10-21

**Authors:** Hai-Xin Liu, Yu-Chang Li, Cai-Xia Liu, Ming-Kuan Yang, Shu-Yuan Zhou, Ai-Ai Dong, Ke Pei, Shi-Yuan Wen

**Affiliations:** 1 College of Traditional Chinese Medicine and Food Engineering, Shanxi University of Chinese Medicine, Taiyuan 030619, China; liuhaixin@sxtcm.edu.cn (H.-X.L.); liucx1104@sxtcm.edu.cn (C.-X.L.); yangmingkuan03@163.com (M.-K.Y.); joooseo_yeon@163.com (S.-Y.Z.); 2 Third Clinical College, Shanxi University of Chinese Medicine, Taiyuan 030619, China; m17636332564@163.com; 3 Second Clinical College, Shanxi University of Chinese Medicine, Taiyuan 030619, China; dongaiai@sxtcm.edu.cn; 4 College of Basic Medical Sciences, Shanxi Medical University, Taiyuan 030600, China

**Keywords:** Shenqi granule, ischemic stroke, uridine metabolism, nucleotide metabolism, GBA, microglia

## Abstract

**Objective:** Shenqi Granules is a traditional Chinese medicine formula derived from the classic prescription Shenqi Formula (SQF), composed of Astragali Radix (Huangqi) and Codonopsis Radix (Dangshen). SQF shows neuroprotection in cerebral ischemia-reperfusion injury (CIRI), but the underlying mechanisms remain unclear. **Methods:** The rat model of middle cerebral artery occlusion (MCAO) was established using the suture method to evaluate the neuroprotective effect of SQF. Blood-absorbed components were identified via UHPLC-Q-TOF-MS/MS, and potential targets and pathways were predicted through network pharmacology. Non-targeted metabolomics of serum and feces was performed using UHPLC-Orbitrap MS, and core metabolic pathways were selected based on regulation magnitude (RM). Gut microbiota structure was analyzed by 16S rRNA sequencing, with associations between microbiota and metabolites assessed via the Mantel test and redundancy analysis (RDA). Weighted gene co-expression network analysis (WGCNA) was applied to screen core Hub genes for ischemic stroke (IS). Based on single-cell RNA data, a uridine transport/metabolism-associated transcriptional signature score (Uridine_Score) was constructed, and cell type-specific coupling between glucocerebrosidase (GBA) and this transcriptional signature was analyzed. Microglial temporal trajectory analysis was conducted using Monocle3. Molecular docking between core components and GBA was verified by AutoDock, and GBA mRNA expression was confirmed by RT-qPCR. **Results:** High-dose SQF reduced infarct volume and neuronal damage and improved behavior. A total of 28 absorbed components and 31 shared IS targets were identified. Metabolomics revealed nucleotide metabolism as a core pathway common to both serum and fecal samples. SQF restored gut–blood uridine balance (serum uridine 0.41× Sham → 0.85× post-SQF), reduced fecal dTMP and hypoxanthine. 16S rRNA sequencing demonstrated that the overall correlation between microbial community structure and nucleotide metabolites was statistically significant (Mantel r = 0.307–0.321, *p* < 0.01). WGCNA highlighted GBA, MMP9, and PYGL. Single-cell analysis showed a microglia-specific uridine transport/metabolism-associated transcriptional signature post-stroke, with GBA most correlated with this signature (ρ = 0.377). Pseudotime revealed microglial state transitions (Homeostatic → Acute_Reactive → DAM), with positive GBA–uridine transport/metabolism-associated transcriptional coupling in the Homeostatic and DAM states. Riboflavin showed the strongest GBA binding (−9.83 kcal/mol), and SQF upregulated GBA mRNA in the penumbra (*p* < 0.0001). **Conclusions:** SQF reprograms nucleotide metabolism via the gut–blood axis and enhances microglial GBA–uridine transport/metabolism-associated transcriptional coupling.

## 1. Introduction

Ischemic stroke (IS), predominantly caused by cerebral ischemia-reperfusion injury (CIRI), represents a leading cause of mortality and long-term disability worldwide [1]. The pathophysiology of CIRI involves a complex cascade of events, including excitotoxicity, oxidative stress, neuroinflammation, and blood–brain barrier disruption, ultimately culminating in neuronal death and irreversible neurological deficits [2]. Despite advances in thrombolytic therapy and endovascular interventions, effective pharmacological strategies to mitigate secondary brain injury and promote functional recovery remain limited [3].

Nucleotide metabolism has emerged as a critical regulatory axis in the pathophysiology of ischemic stroke [4]. As essential building blocks of nucleic acids and key energy carriers, nucleotides participate in diverse cellular processes, including ATP generation, signal transduction, and epigenetic modulation [5]. Beyond their canonical roles in energy provision, nucleotides function as extracellular signaling molecules modulating neuroinflammatory responses [6]. Uridine is a core molecule in the nucleotide metabolic network. It serves both as a key precursor for the synthesis of uridine monophosphate (UMP) and participates in the broader nucleotide pool balance through its degradation products [7]. In animal models of ischemic stroke, oral administration of uridine significantly reduced cerebral infarct volume in mice subjected to middle cerebral artery occlusion (MCAO) [8]. One study further demonstrated that a specific nutrient combination containing uridine, choline, and multiple vitamins alleviated neuroinflammation and improved brain function and motor performance in post-stroke mice [9]. In addition, uridine has been shown to protect neurons against cell death induced by energy deprivation (e.g., glucose deprivation), with the underlying mechanism potentially involving the maintenance of intracellular ATP levels [8].

Traditional Chinese medicine (TCM) has garnered increasing attention as a promising therapeutic approach for IS, offering multi-target and multi-pathway intervention advantages [10,11]. Shenqi Granules, derived from the ancient classic prescription Shenqi Formula (SQF), is a classical TCM prescription comprising Astragali Radix (Huangqi) and Codonopsis Radix (Dangshen) [12]. Our previous study demonstrated that SQF exhibits notable efficacy in promoting neurological functional recovery and attenuating cerebral infarct volume in experimental stroke models [13]. Importantly, SQF promotes CIRI repair by modulating tryptophan and tyrosine metabolism, elucidating its therapeutic mechanism solely from the perspective of gut metabolism and microbiota. However, the bioactive constituents of SQF that enter systemic circulation and their direct contributions to CIRI recovery remain unexplored. Therefore, the present study focuses on identifying SQF components absorbed into the bloodstream, aiming to comprehensively investigate the mechanisms underlying SQF-mediated amelioration of IS.

## 2. Materials and Methods

### 2.1. Drugs and Animals

Shenqi Granules, derived from the ancient classic prescription Shenqi Formula (SQF), were sourced from Taiji Group Co., Ltd. (Chengdu, China). Nimodipine, employed as the positive control, was procured from MedChemExpress (HY-B0265, Shanghai, China).

Eight-week-old male SD rats (250–280 g) were supplied by Vital River Laboratory Animal Technology Co. (Beijing, China). After arrival, the animals were housed for 7 days at 25 ± 2 °C and 60 ± 5% relative humidity under a 12 h light/dark cycle with ad libitum food and water. All procedures followed the “Guidelines for the Care and Use of Laboratory Animals” and were approved by the Animal Ethics Committee of Shanxi University of Chinese Medicine (approval No. AWE202603176).

### 2.2. CIRI Animal Model and Administration Regimen

CIRI was induced by transient middle cerebral artery occlusion (MCAO) in Sprague Dawley rats. Following a 12 h fasting period, animals were anesthetized with intraperitoneal sodium pentobarbital. The right common carotid artery (CCA), external carotid artery (ECA), and internal carotid artery (ICA) were carefully exposed through a midline neck incision. The ECA was ligated distally with a loose knot, while the CCA was permanently ligated proximally. Temporary occlusion of the ICA was achieved using a microvascular clip. A small arteriotomy was performed on the CCA between the two ligatures, through which a nylon filament (rounded tip, diameter 0.26–0.28 mm) was advanced intraluminally into the ICA and further cranially until resistance was felt (approximately 18–20 mm from the carotid bifurcation), thereby blocking the origin of the middle cerebral artery. After 2 h of ischemia, the filament was gently withdrawn to restore cerebral blood flow. The CCA was permanently ligated distal to the incision site, and blood supply through the ECA was re-established by releasing its ligature. The incision was closed in layers, the wound margin was sterilized, and prophylactic penicillin was administered. Sham-operated animals underwent identical surgical exposure of the cervical vasculature without arterial occlusion or filament insertion.

Rats were randomly allocated into five experimental cohorts (*n* = 6 per group): (i) sham-operated control receiving vehicle; (ii) MCAO model receiving vehicle; (iii) MCAO with nimodipine reference treatment (18 mg/kg, i.g.); (iv) MCAO with low-dose SQF intervention (1.5 g/kg, i.g.); and (v) MCAO with high-dose SQF intervention (3.0 g/kg, i.g.) according to our previous research [13]. Pharmacological administration commenced 2 h post-reperfusion and continued once daily for 14 consecutive days. The vehicle consisted of 0.5% carboxymethylcellulose sodium suspension in distilled water, administered at equivalent volumes across all groups. A total of 30 male SD rats were finally included in this study, with 6 rats in each group. The overall success rate of MCAO modeling was approximately 75%, and only rats with a Longa score of 1–3 were considered successfully modeled and included in the final analysis. Rats that died before the scheduled endpoint or had no neurological deficit were excluded. MCAO model establishment was confirmed using the Longa score, a standardized 5-point scale for assessing neurological deficits in rodents.

### 2.3. Animal Behavior

An open field test was performed to assess locomotor activity and anxiety-like behavior. Animals are placed in the center of a square arena (typically 50 cm × 50 cm or 100 cm × 100 cm with 30–40 cm high walls) and allowed to explore freely for 5–10 min. The arena is divided into central and peripheral zones, and behaviors are recorded using video tracking software. Key parameters include total distance traveled (locomotor activity), time spent in the center versus periphery (anxiety-like behavior), and frequency of rearing and grooming (exploratory behavior).

The novel object recognition test consists of three phases: habituation (5–10 min in an empty open field), training (5–10 min exposure to two identical objects), and testing (5 min with one familiar and one novel object following a retention delay of 1–24 h). The discrimination index is calculated as (time spent with novel object − time spent with familiar object)/total exploration time, with values above zero indicating intact recognition memory. The test requires no external motivation such as food restriction or aversive stimuli.

The Morris water maze (MWM) was performed. Animals are trained to locate a submerged hidden platform in a circular pool (100–180 cm diameter) filled with opaque water maintained at 22 ± 1 °C. During the 4- to 5-day acquisition phase, animals receive four daily trials with randomized start positions, and escape latency and path length are recorded. A probe trial (platform removed) is conducted on day 6 to assess spatial memory retention by measuring time spent in the target quadrant and platform crossing frequency.

### 2.4. Nissl Staining

Brain tissues were fixed in 4% paraformaldehyde, embedded in paraffin, and sectioned coronally at 5 μm thickness. After deparaffinization and rehydration, sections were stained with 0.5% cresyl violet acetate for 5–10 min, differentiated in 95% ethanol, dehydrated, cleared in xylene, and mounted with neutral balsam. Nissl-positive neurons displaying intact morphology with distinct nucleoli were counted in the ischemic penumbra under a light microscope at 400× magnification by an investigator blinded to group allocation.

### 2.5. 2,3,5-Triphenyltetrazolium Chloride (TTC) Staining

Brains were harvested after the intervention ended, rapidly frozen at −20 °C for 10 min, and sliced into 2 mm coronal sections using a brain matrix. Sections were incubated in 2% TTC solution dissolved in phosphate buffer (pH 7.4) at 37 °C for 20 min in the dark, followed by fixation in 4% paraformaldehyde overnight. Viable tissue stained red, while infarcted areas remained pale white. Images were captured with a digital camera (EOS R, Canon, Tokyo, Japan) and analyzed using ImageJ software (v1.54r) to calculate infarct volume, with edema correction applied using the following formula: corrected infarct volume = left hemisphere volume − (right hemisphere volume − measured infarct volume).

### 2.6. Identification of Blood-Entering SQF Components

Serum samples were collected from SQF-treated CIRI rats at designated time points post-administration. Blood was drawn from the abdominal aorta, allowed to clot at room temperature for 30 min, and centrifuged at 3000× *g* for 15 min to obtain serum. The supernatant was transferred to a clean tube and stored at −80 °C until analysis.

For serum pharmacochemistry analysis, an aliquot of serum was mixed with three volumes of methanol, vortexed for 3 min, and centrifuged at 12,000× *g* for 10 min to precipitate proteins. The supernatant was evaporated to dryness under nitrogen and reconstituted in the initial mobile phase. UHPLC-Q-TOF-MS analysis was performed in both positive and negative ionization modes. Chromatographic separation was achieved on a C18 column (2.1 mm × 100 mm, 1.8 μm) with a gradient elution of acetonitrile and 0.1% formic acid in water. The mass spectrometer (X500 QTOF; SCIEX, Framingham, MA, USA) was operated in information-dependent acquisition (IDA) mode. A full-scan survey (MS1) was acquired over *m*/*z* 100–1500, and collision-induced dissociation (CID) was used to acquire MS/MS spectra of selected precursor ions (*m*/*z* 50–1500).

Raw data were processed using SCIEX OS software (v3.3.0; SCIEX, Framingham, MA, USA) for peak extraction and alignment. Mass spectra were matched against in-house databases of SQF constituents, reference standards, and reported metabolites. Compounds detected in drug-containing serum but absent in blank serum and sham-operated controls were defined as prototype components and metabolites entering circulation, respectively. Structural identification was confirmed by comparing retention times, accurate molecular masses, fragment ion patterns, and isotopic distributions with authentic standards or literature data.

### 2.7. Untargeted Metabolomic Analysis

Fecal and serum samples were collected at the experimental endpoint, snap-frozen in liquid nitrogen, and stored at −80 °C until analysis. Metabolite extraction was performed using methanol–acetonitrile–water (2:2:1, *v*/*v*/*v*) for feces and methanol for serum, followed by vortexing, sonication, and centrifugation. The supernatants were subjected to ultra-high-performance liquid chromatography coupled with Orbitrap mass spectrometry (UHPLC-Orbitrap MS;Vanquish UHPLC system coupled to an Orbitrap Exploris™ 480 mass spectrometer, Thermo Fisher Scientific, Waltham, MA, USA) in both positive and negative electrospray ionization modes. Chromatographic separation was achieved on an ACQUITY UPLC HSS T3 column (2.1 mm × 100 mm, 1.8 μm; Waters Corp., Milford, MA, USA) with a gradient mobile phase of 0.1% formic acid in water and acetonitrile. Raw MS data were processed with Progenesis QI software (v3.1, Waters Corp., Milford, MA, USA) for peak alignment, annotation, and normalization. Multivariate statistical analyses, including principal component analysis (PCA), were conducted to identify differential metabolites. Metabolites with variable importance in projection (VIP) > 1 and *p* < 0.05 were considered significantly altered and subsequently mapped to metabolic pathways using the KEGG database.

### 2.8. 16S rRNA Gene Sequencing Analysis

Fecal samples were collected under sterile conditions, immediately flash-frozen in liquid nitrogen, and stored at −80 °C until DNA extraction. Total bacterial genomic DNA was extracted using a commercial stool DNA kit (D5625-01; Omega Bio-Tek, Norcross, GA, USA) according to the manufacturer’s protocol, with bead-beating homogenization to ensure efficient lysis of Gram-positive bacteria. The V3–V4 hypervariable regions of the 16S rRNA gene were amplified using universal primers 338F (5′-ACTCCTACGGGAGGCAGCAG-3′) and 806R (5′-GGACTACHVGGGTWTCTAAT-3′). Amplicons were purified with AMPure XP beads (Beckman Coulter, Brea, CA, USA), quantified by a Qubit fluorometer (Thermo Fisher Scientific, Waltham, MA, USA), and pooled for paired-end sequencing (2 × 300 bp) on the Illumina MiSeq platform (Illumina, San Diego, CA, USA). Raw reads were demultiplexed, quality-filtered, and merged using the QIIME2 (v2021.11) pipeline. Chimeric sequences were removed, and amplicon sequence variants (ASVs) were clustered at a 100% similarity threshold. Taxonomic classification was assigned against the SILVA (version 138). Alpha diversity (Chao1, Shannon, Simpson indices) and beta diversity (PCoA based on Bray-Curtis and Unweighted UniFrac distances) were calculated, with differential abundance analysis performed using LEfSe (v1.1.2) and DESeq2 (v1.26.0) (LDA score > 2, *p* < 0.05).

### 2.9. Target Prediction of Blood-Absorbed SQF Components

The 28 blood-absorbed prototype components and metabolites identified by UHPLC-Q-TOF-MS/MS in Section 2.6 were used for target prediction. Chemical structures were retrieved from PubChem and converted to canonical SMILES format. Potential targets were predicted using SwissTargetPrediction (http://www.swisstargetprediction.ch, accessed on 10 October 2025). Predicted targets from all compounds were consolidated, standardized using UniProt IDs, and merged to generate the SQF intervention target set. Redundant entries were removed. This target set was used for subsequent network pharmacology, WGCNA intersection, and KEGG pathway enrichment analyses.

### 2.10. Associated Targets of IS

IS-related datasets GSE16561 and GSE58294 were retrieved from GEO and merged using inSilicoMerging with empirical Bayesian batch correction. Data preprocessing excluded genes and samples with >50% missing values, followed by KNN imputation and Z-score normalization. Differential expression analysis was performed using limma to identify IS-related DEGs. The intersection of DEGs and SQF component targets was subjected to KEGG pathway enrichment analysis using clusterProfiler (min gene set = 5, max = 5000, *p* < 0.05), with visualization by pathview.

### 2.11. Cross-Species Ortholog Mapping

To integrate transcriptomic data across species, human genes from the merged bulk RNA-seq datasets (GSE16561 and GSE58294) were mapped to their rat (Rattus norvegicus) and mouse (Mus musculus) orthologs using Ensembl BioMart (GRCh37, release 75, https://grch37.ensembl.org, (accessed on 28 September 2026)). The BioMart “Homology” dataset was queried to retrieve one-to-one orthologs across human, rat, and mouse. Only genes with unambiguous one-to-one ortholog assignments were retained for downstream integration. Genes with many-to-many, one-to-many/many-to-one, or missing ortholog mappings were excluded.

### 2.12. Weighted Gene Co-Expression Network Analysis (WGCNA)

WGCNA was performed using the WGCNA package (v1.72-5) in R (seed = 12345). Genes with variance above the 75th percentile were retained after quality control and outlier removal. A signed network was constructed with optimal soft threshold selection, and modules were identified by dynamic tree cutting (minimum size = 50) with a merging threshold of 0.2. Module–trait correlations were calculated, and hub genes were identified from the green, magenta, and turquoise modules based on module membership (|MM| ≥ 0.8) and gene significance (GS ≥ 0.05), with reliability verified by MM-GS scatter plots.

### 2.13. Single-Cell RNA Sequencing (scRNA-Seq)

ScRNA-seq data from GSE225948 in the GEO database was used, including three experimental groups: sham operation group (Sham), 2 days post-stroke group (Stroke-D2), and 14 days post-stroke group (Stroke-D14). After data reading, cells with nCount RNA ≥ 200 and nFeature RNA ≥ 100 were retained; cells with mitochondrial gene proportion ≤ 10% were retained; cells with ribosomal protein gene (Rpl/Rps family) proportion ≤ 60% were retained; and the 95th percentile of nFeature RNA was used as the upper screening limit to filter outliers. Quality control effects were displayed through violin plots and scatter plots (nCount RNA vs. nFeature RNA correlation). Doublet detection was performed using the scDblFinder package, with doublet determination threshold set to 0.2 and feature gene number set to 1500. Removal effects were verified through QC feature distribution comparison and nCount/nFeature scatter plots.

Single-cell data were normalized using SCTransform (v2, glmGamPoi method) independently for each experimental group. Integration was performed via the SCT anchor method: 3000 highly variable genes were selected as features, intergroup anchors were identified, and an integrated assay was generated. Dimensionality reduction was conducted using the first 30 principal components, followed by UMAP visualization. Clustering was performed via SNN graph construction at resolution 0.8. Optimal PCA dimensions were determined by scree plots, and clustering quality was verified by UMAP visualization to confirm effective batch effect removal and distinct cell type separation.

Referencing single-cell sequencing literature [14,15,16,17,18], a brain tissue-specific Marker gene set covering 18 cell types was screened. ScaleData normalization was performed on SCT assay data. For Marker scoring calculation, for each cell type, MarkerScore was calculated based on the expression difference between its core Marker genes and control genes, matching the corresponding cluster. After annotation, cell type distribution was visualized through UMAP plots, and annotation accuracy was verified through Marker gene expression heatmaps.

Single-cell transcriptomic data from a previously annotated mouse ischemic stroke dataset were utilized, comprising three experimental groups: Sham, stroke day 2 (Stroke-D2), and stroke day 14 (Stroke-D14). Quality control and normalization were performed following the standard Seurat (v5.4.0) workflow, and the RNA count matrix was retained for downstream analyses. Cell types were identified based on canonical marker genes, including microglia, macrophages, endothelial cells, neutrophils, NK cells, B cells, oligodendrocytes, and immature neurons. Batch information (GSM identifiers) was extracted from cell barcodes and incorporated as a random effect in mixed models to correct for batch variation.

### 2.14. Cell Type-Specific Uridine Consumption Assessed by Linear Mixed Model

To quantify uridine metabolic status at the single-cell level, multi-level gene modules were constructed based on the KEGG pyrimidine metabolism pathway (hsa00240). The core consumption module (Uridine_Core) included key catabolic enzymes such as uridine phosphorylases (*Upp1*, *Upp2*), uridine-cytidine kinases (*Uck1*, *Uck2*, *Uckl1*), thymidine kinases (Tk1, Tk2), deoxycytidine kinase (Dck), and thymidine phosphorylase (Tymp). The nucleoside transport module (Nucleoside_Transport) comprised equilibrative nucleoside transporters (Slc29a1-3) and concentrative nucleoside transporters (Slc28a1-3).

Module activity scores were calculated using the UCell algorithm (maxRank = 150). Principal component analysis (PCA) was applied to the UCell scores of both modules, and the first principal component (PC1) was reverse-scored (multiplied by −1) so that higher values corresponded to greater uridine uptake and catabolic activity, defined as the Uridine Comprehensive Score (Uridine_Score). The original PC1 loadings indicated coordinated changes between uridine catabolic enzymes and nucleoside transporters, and after reverse scoring, higher values represented a stronger “uridine-consuming” phenotype. This score was z-score-standardized to Uridine_Score_z for subsequent modeling. Additionally, the AUCell algorithm was independently employed to calculate the same module scores for cross-validation, with results stored in the Seurat object.

A pseudo-bulk strategy was adopted, wherein the mean Uridine_Score was computed for each sample–cell type combination. A linear mixed model (LMM) was constructed as follows: Mean score ~ group × Annotated CellType + (1|sample id). The main effects and interaction effects were evaluated using Type II Wald chi-square tests. Post hoc multiple comparisons were performed with the emmeans package, and *p*-values were adjusted using the Benjamini–Hochberg (BH) method.

For each cell type, Spearman rank correlation coefficients between each gene and Uridine_Score were calculated, and genes were ranked in descending order of correlation coefficient. Enrichment of the KEGG_Pyrimidine_Metabolism pathway was tested using the fgsea package, with parameters set to minSize = 5 and maxSize = 500. |NES| > 1 and adjusted *p*-value (*p*adj) < 0.05 were considered statistically significant.

### 2.15. Multi-Level Analysis of GBA–Uridine Metabolic Coupling

GBA expression was rank-based inverse normal transformed to obtain GBA_rank. For each cell type, mixed models Uridine_Score ~ (1|sample_id) and GBA_rank ~ (1|sample_id) were fitted separately, residuals were extracted, and Spearman rank correlation coefficients were computed with BH correction. For cell types with fewer than three samples, the model was simplified to a linear model for residual extraction. For each cell type, two nested models were compared: Full model: GBA_rank ~ Uridine_Score_z × group + (1|sample_id); and Main-effects model: GBA_rank ~ Uridine_Score_z + group + (1|sample_id).

Significance of the interaction term was assessed using likelihood ratio tests (LRTs), and ΔAIC (AIC_main − AIC_full) was calculated. Only cell types with ≥ 30 cells, presence in all three groups, and at least two independent samples were included in this analysis.

The scMetabolism package was used to compute KEGG pyrimidine and purine metabolism pathway activities based on AUCell. Spearman correlation between Uridine_Score and pyrimidine metabolism scores was calculated as a measure of convergent validity. To evaluate metabolic specificity, the interaction effect between GBA_rank and purine metabolism activity was further tested using the model GBA_rank ~ scMetab_Purine_metabolism × group + (1|sample_id).

All analyses were conducted in R v4.5.1. Correlation analyses were performed using Spearman’s rank correlation. Mixed models were fitted using the lme4 and lmerTest packages with the bobyqa optimizer. Multiple testing corrections were uniformly applied using the BH method to control the false discovery rate.

### 2.16. Microglia Subsetting and Subtype Characterization

Microglia were subsetted from the previously constructed Seurat object of the single-cell transcriptomic atlas of IS. Variance-stabilizing transformation (vst) was applied to select the top 2000 highly variable genes, with regression correction for mitochondrial gene percentage (percent.mt) and RNA count (nCount_RNA). Principal component analysis (PCA, npcs = 50) and uniform manifold approximation and projection (UMAP, dims = 1:30, spread = 2.5, min.dist = 0.8, n.neighbors = 20) were subsequently performed. A shared nearest neighbor graph was constructed based on the first 30 principal components, and graph-based clustering was conducted using the Louvain algorithm (FindClusters, resolution = 0.5). Differentially expressed genes between clusters were identified using the Wilcoxon rank-sum test (FindAllMarkers, retaining only positive markers with min.pct ≥ 0.25, logFC ≥ 0.25, and adjusted *p* < 0.05). The top 10 marker genes per cluster, ranked by average log2 fold change, were extracted for annotation. Significantly differentially expressed genes from each cluster were converted to ENTREZ IDs and subjected to Gene Ontology biological process (GO-BP) and KEGG pathway enrichment analyses using clusterProfiler, with BH correction and significance thresholds set at adjusted *p* < 0.05 and q < 0.05.

A two-stage annotation strategy was employed. In the first stage, a UCell score was calculated based on the stroke-associated microglia (SAM) signature [19], using the gene set comprising *Spp1*, *Fabp5*, *Gpnmb*, *Ctsb*, *Ctsl*, *Lgals3*, *Lpl*, *Fth1*, *Cd63*, and *Ctsd* (maxRank = 150). In the second stage, integrating marker gene expression, GO/KEGG functional characteristics, and SAM scores, the eight clusters were annotated into five functional subtypes: Homeostatic, Acute_Reactive, disease-associated microglia (DAM), Proliferating, and Infiltrating macrophages (Infiltrating_Mφ).

Given the highly skewed distributions of Uridine_Score and GBA_rank, heatmap visualization was performed using mean normalization, and intergroup differences were assessed by Dunn’s test (dunn.test, BH-corrected) with Homeostatic as the reference. Compositional differences across subtypes were evaluated using Pearson’s chi-square test. To eliminate batch effects across samples, a linear mixed-effects model (LMM, lme4) with random intercept (1|sample_id) was fitted within each subtype; for subtypes with fewer than three samples, a simple linear model was used instead. Residuals were extracted from the models, and Spearman’s rank correlation test was applied to evaluate the independent association between GBA and uridine, with BH correction. Interaction effects were examined by fitting the model ‘GBA_rank ~ Uridine_Score × group + (1|sample_id) ‘ within each subtype, compared with a main-effects-only model via likelihood ratio tests (LRTs); interaction term coefficients were extracted and corrected.

Pseudotime analysis was conducted using Monocle3 (v1.x). The Seurat object was converted to CellDataSet format, followed by PCA preprocessing with 30 principal components retained. UMAP dimensionality reduction and cell clustering (resolution = 0.0001) were performed based on the PCA results. A principal trajectory graph was constructed using ‘learn_graph()’. The root node was defined by referencing the spatial center of Sham group cells in UMAP space, mapped to the nearest principal node (Y_10), from which pseudotime for each cell was calculated via ‘order_cells()’.

Based on the previously constructed uridine metabolic gene modules, Uridine_Score was calculated, and GBA expression rank (GBA_rank) was extracted. Spearman’s rank correlation coefficients were used to assess the association and significance of these metrics with pseudotime.

Nine representative genes (*P2ry12*, *Tmem119*, *Spp1*, *Apoe*, *Clec7a*, *Top2a*, *Ccr2*, *Gba1*, and *Upp1*) were selected, and their log-normalized expression values were extracted. Scatter plots with LOESS smoothing curves (span = 0.3) were generated using ggplot2, with pseudotime as the *x*-axis and faceted by cell subtype.

Gene Ontology (GO) biological process enrichment analysis was performed for branch-dependent genes. Gene symbols were converted to ENTREZ IDs using clusterProfiler, and enrichment was conducted via ‘enrichGO()’ (OrgDb = org.Mm.eg.db, pAdjustMethod = “BH”, qvalueCutoff = 0.05). The top 15 significantly enriched terms were visualized as dot plots.

### 2.17. Molecular Docking

Molecular docking was performed using AutoDock 4.2.6 to predict the binding interactions between the core target GBA and the SQF-derived blood-absorbed components and metabolites. The crystal structure of human GBA was retrieved from the RCSB Protein Data Bank (PDB ID: 6TN1). The receptor was prepared using AutoDockTools by removing water molecules and adding polar hydrogens according to standard procedures. A total of eight unique compounds were selected for docking, including SQF-derived ingredients (uridine, riboflavin, and formononetin-7-glucoside) and SQF-related nucleotide metabolites (deoxyinosine, inosine, uridine, xanthosine, 2′-deoxyinosine, and adenosine). The three-dimensional structures of these ligands were obtained from PubChem and energy-minimized prior to docking. For each receptor–ligand pair, ten independent docking runs were performed. The docking results were clustered, and the binding energies of the different conformational clusters were obtained. The best-scoring conformation was selected to represent the binding mode between each ligand and GBA.

### 2.18. Reverse Transcriptase Quantitative Polymerase Chain Reaction (RT-qPCR)

Total RNA was isolated from rat brain tissues using TRIzol reagent, followed by reverse transcription into cDNA with a reverse transcription kit. RT-qPCR was performed using SYBR Green PCR Master Mix on a real-time PCR system. The relative mRNA expression level of *Gba1* was normalized to the housekeeping gene Gapdh and calculated using the 2^−ΔΔCt^ method.

### 2.19. Statistical Analysis

Statistical analysis was performed using GraphPad Prism (version 9.0) or R software (version 4.5.1). Data are presented as mean ± standard deviation (SD) or median with interquartile range, depending on distribution normality assessed by the Shapiro–Wilk test. Comparisons between two groups were analyzed using the unpaired Student’s *t*-test or the Mann–Whitney U test. Multiple group comparisons were conducted using one-way analysis of variance (ANOVA) followed by Tukey’s post hoc test for normally distributed data, or the Kruskal–Wallis test with Dunn’s post hoc correction for non-parametric data. Two-way ANOVA was applied for analyses involving two independent variables. Repeated measures ANOVA was used for longitudinal data such as Morris water maze training trials. Statistical significance was set at *p* < 0.05.

## 3. Results

### 3.1. SQF Ameliorates CIRI and Nerve Damage

**Figure 1:**
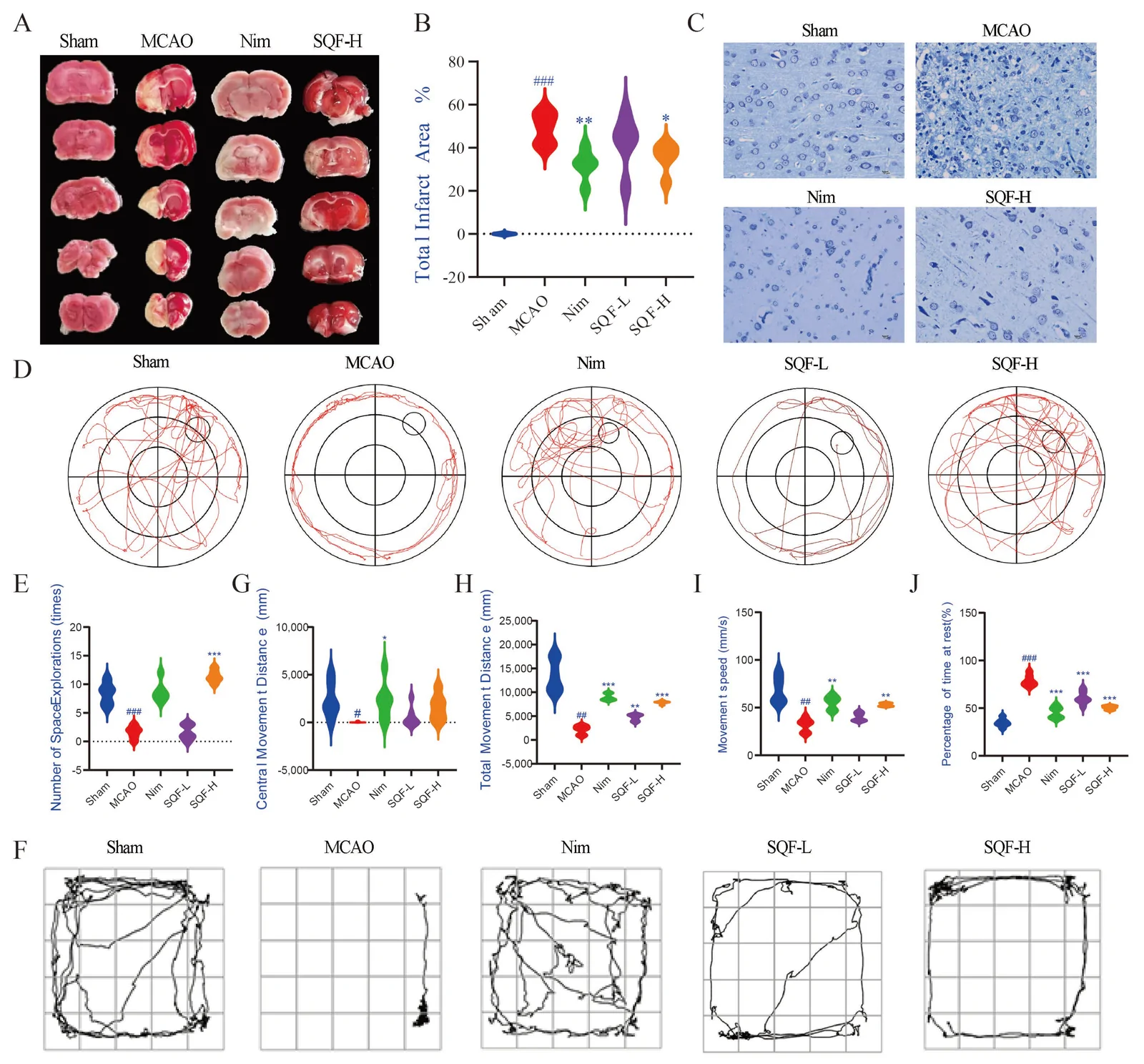
Neuroprotective effects of SQF on CIRI in rats. (**A**) Representative TTC-stained brain sections showing infarct areas (pale) in Sham, MCAO, Nimodipine (Nim), and SQF-treated groups. (**B**) Quantification of total infarct area percentage. (**C**) Representative Nissl-stained brain sections showing histopathological changes. (**D**) MWM probe trial: representative swimming path traces. (**E**) Number of platform location crossings in the MWM probe trial. (**F**) Open field test: representative locomotion traces. (**G**–**J**) Open field test parameters: central movement distance (**G**), total movement distance (**H**), movement speed (**I**), and percentage of time at rest (**J**). # *p* < 0.05, ## *p* < 0.01, ### *p* < 0.001, compared with the Sham group. * *p* < 0.05, ** *p* < 0.01, *** *p* < 0.001, compared with the MCAO group.

Compared with the Sham group, the MCAO group exhibited a significantly increased cerebral infarct area (Figure 1A,B), accompanied by severe histopathological damage (Figure 1C), impaired spatial memory (Figure 1D,E), and reduced locomotor activity (Figure 1F–J), as evidenced by a higher percentage of time at rest, shorter total movement distance, slower speed, and fewer central zone explorations. Treatment with nimodipine (Nim) or SQF markedly alleviated these ischemia-induced deficits. Specifically, both treatments significantly reduced the infarct area (Figure 1B), improved neuronal morphology (Figure 1C), and restored spatial memory (Figure 1D), as shown by increased platform crossings in the MWM probe trial (Figure 1E). In the open field test (Figure 1F), Nim and SQF treatment significantly increased central movement distance (Figure 1G), total movement distance (Figure 1H), and movement speed (Figure 1I) while decreasing the time at rest (Figure 1J), indicating improved locomotor function and reduced anxiety-like behavior. Notably, SQF treatment showed comparable or superior neuroprotective effects relative to nimodipine, particularly in enhancing spatial exploration and central zone activity (Figure 1E,G). Therefore, SQF ameliorates CIRI and nerve damage.

### 3.2. Blood-Entering Components and Targets of SQF for CIRI Improvement

**Figure 2:**
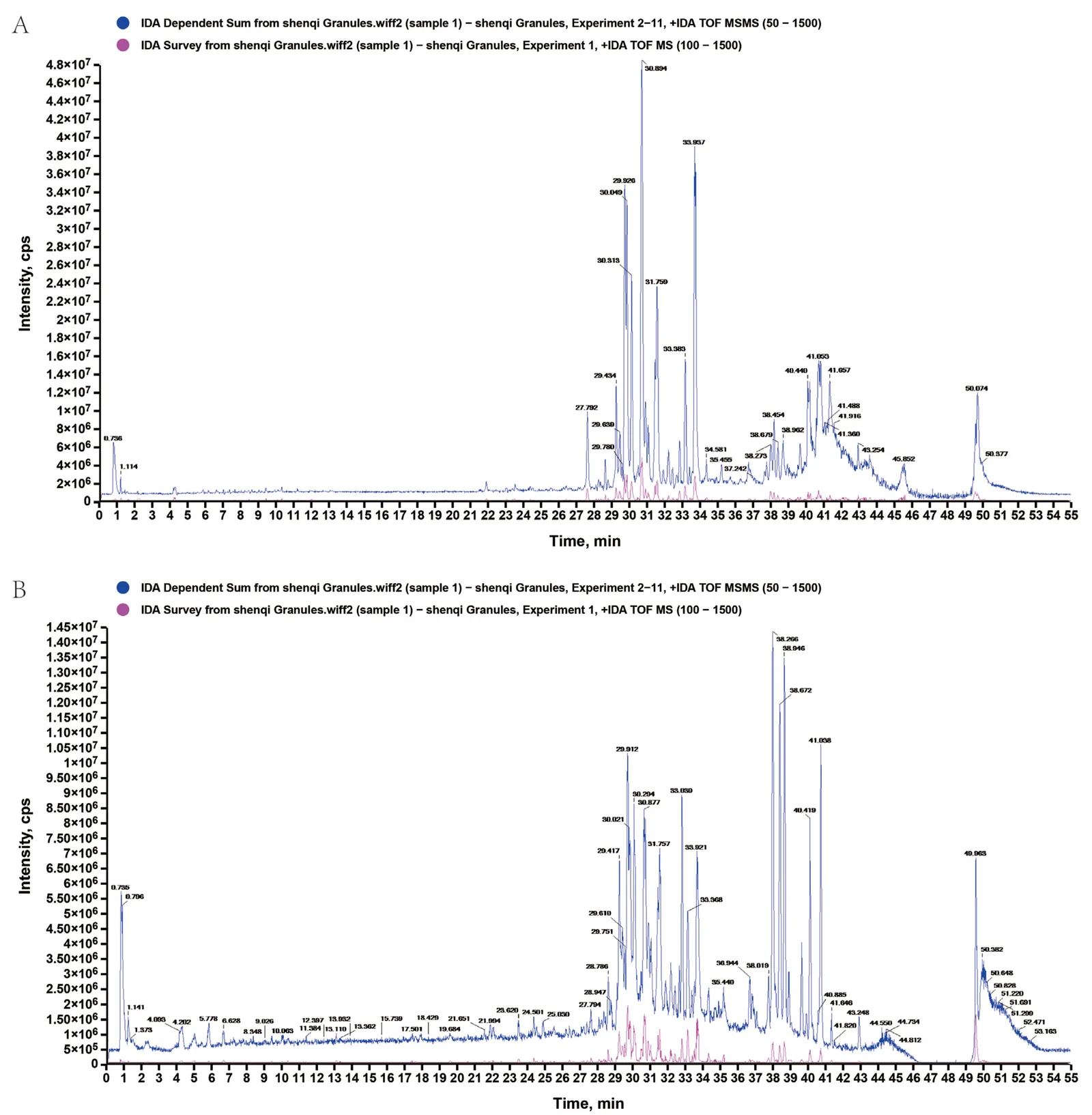
Blood-entering components of SQF. (**A**,**B**) Representative total ion chromatograms (TICs) of SQF blood-entry ingredients analyzed by UHPLC-Q-TOF-MS/MS, showing compound separation and peak identification in positive (**A**) and negative (**B**) ion modes.

**Figure 3:**
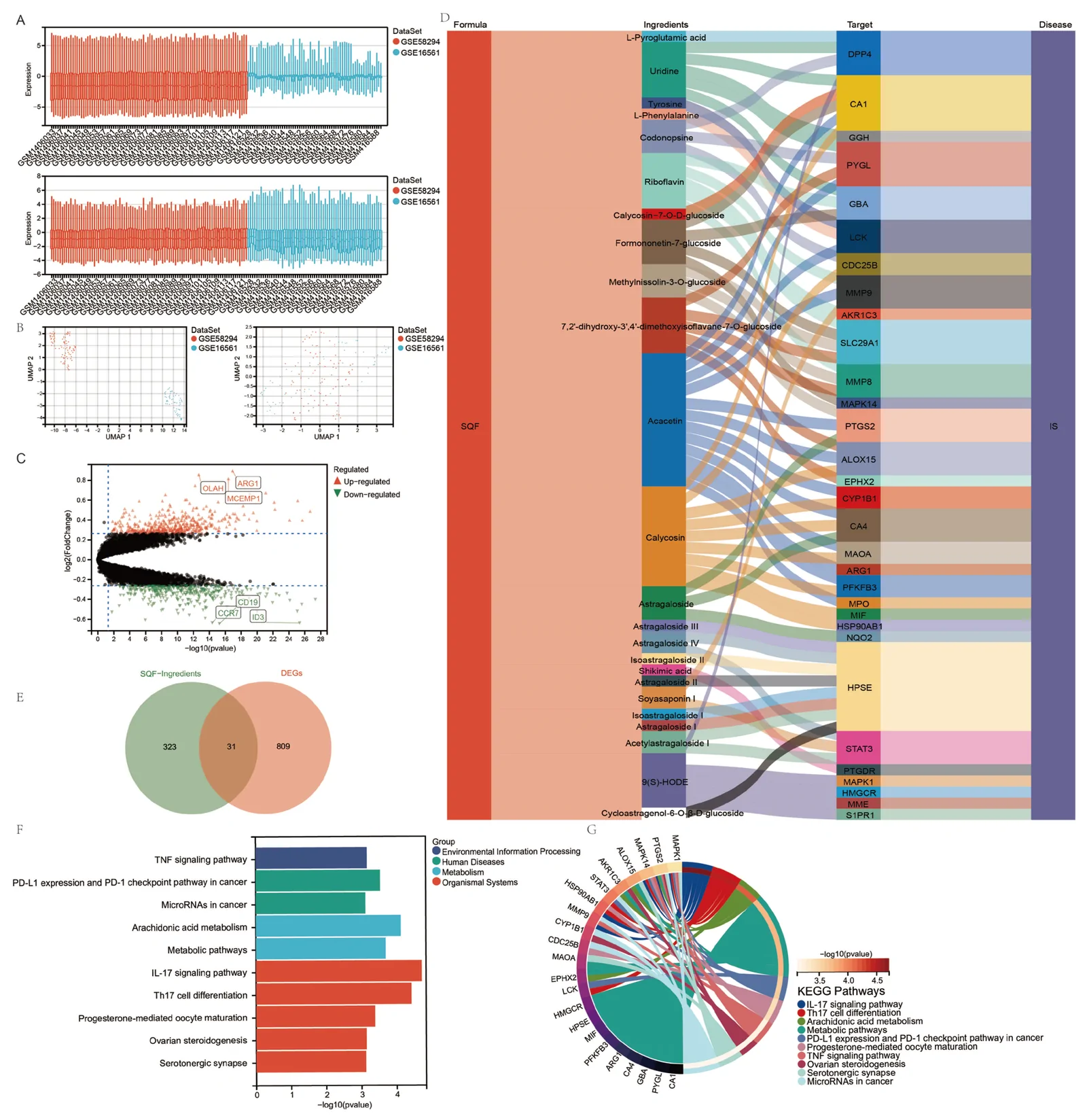
Targets of SQF for CIRI improvement. (**A**) Expression heatmaps of top DEGs from two datasets (GSE58294, red; GSE16561, teal), illustrating distinct gene expression profiles between groups. (**B**) UMAP dimensionality reduction plots showing clear separation of samples from GSE58294 and GSE16561 datasets, confirming dataset-specific transcriptomic patterns. (**C**) Volcano plot of DEGs, highlighting significantly upregulated (red triangles) and downregulated (green triangles) genes. (**D**) Sankey diagram of the formula–ingredient–target–disease network for SQF against IS. Ribbon width represents interaction frequency. (**E**) Venn diagram showing the overlap (31 targets) between targets of SQF blood-entry components and DEGs, identifying potential key targets for SQF neuroprotection. (**F**) Bar plot of top-enriched KEGG pathways. *X*-axis: −log_10_(*p*-value). Pathways are grouped by functional categories. (**G**) Gene-pathway interaction Circos plot. Color gradient denotes enrichment significance (−log_10_(*p*-value)).

UHPLC-Q-TOF-MS/MS analysis identified the systemic absorption components of SQF in positive (Figure 2A) and negative (Figure 2B) ion modes, revealing a complex profile of bioactive constituents with efficient chromatographic separation (Table 1). Among the 28 blood-absorbed components, uridine, riboflavin, formononetin-7-glucoside, calycosin, astragaloside IV, and codonopsine were selected as key components for subsequent target prediction and molecular docking based on their reported bioactivities and/or relevance to nucleotide metabolism. Because SQF consists only of Astragali Radix and Codonopsis Radix, the identified flavonoids (e.g., calycosin and formononetin-7-glucoside) and astragalosides (e.g., astragaloside IV) are consistent with characteristic constituents of Astragali Radix, whereas codonopsine is a characteristic alkaloid of Codonopsis Radix. Other components, including amino acids, nucleobases, riboflavin, shikimic acid, and 9(S)-HODE, are widely distributed and are therefore not unique to SQF; however, they may still be derived from these two herbs. The WD label does not exclude the possibility that these components originate from Astragali Radix or Codonopsis Radix; it only indicates that they cannot serve as SQF-specific markers. Transcriptomic analysis of two IS datasets (GSE58294/GSE16561) demonstrated distinct gene expression patterns via heatmap clustering (Figure 3A) and UMAP separation (Figure 3B). Volcano plot analysis identified key dysregulated genes in ischemia, including upregulated and downregulated candidates (Figure 3C). The Sankey diagram was constructed to visualize the formula–ingredient–target–disease network, which demonstrated that SQF exerted therapeutic effects against IS via multiple bioactive compounds acting on diverse target genes, indicating a multi-component and multi-target feature (Figure 3D). Venn-diagram analysis was performed to screen hub genes by intersecting SQF-related targets and disease-derived DEGs. A total of 31 overlapping genes were identified as the potential core targets of SQF for IS treatment (Figure 3E). KEGG pathway enrichment analysis was conducted based on these 31 hub targets. The bar chart revealed significantly enriched pathways, including the TNF signaling pathway, PD-L1 expression and PD-1 checkpoint pathway in cancer, the IL-17 signaling pathway, and arachidonic acid metabolism (Figure 3F). The gene-pathway Circos plot further illustrated the interaction relationships between hub genes and enriched pathways, suggesting that SQF might ameliorate IS through the regulation of inflammation- and immune-related signaling pathways (Figure 3G).

### 3.3. SQF Improves CIRI by Regulating Nucleotide Metabolism

**Figure 4:**
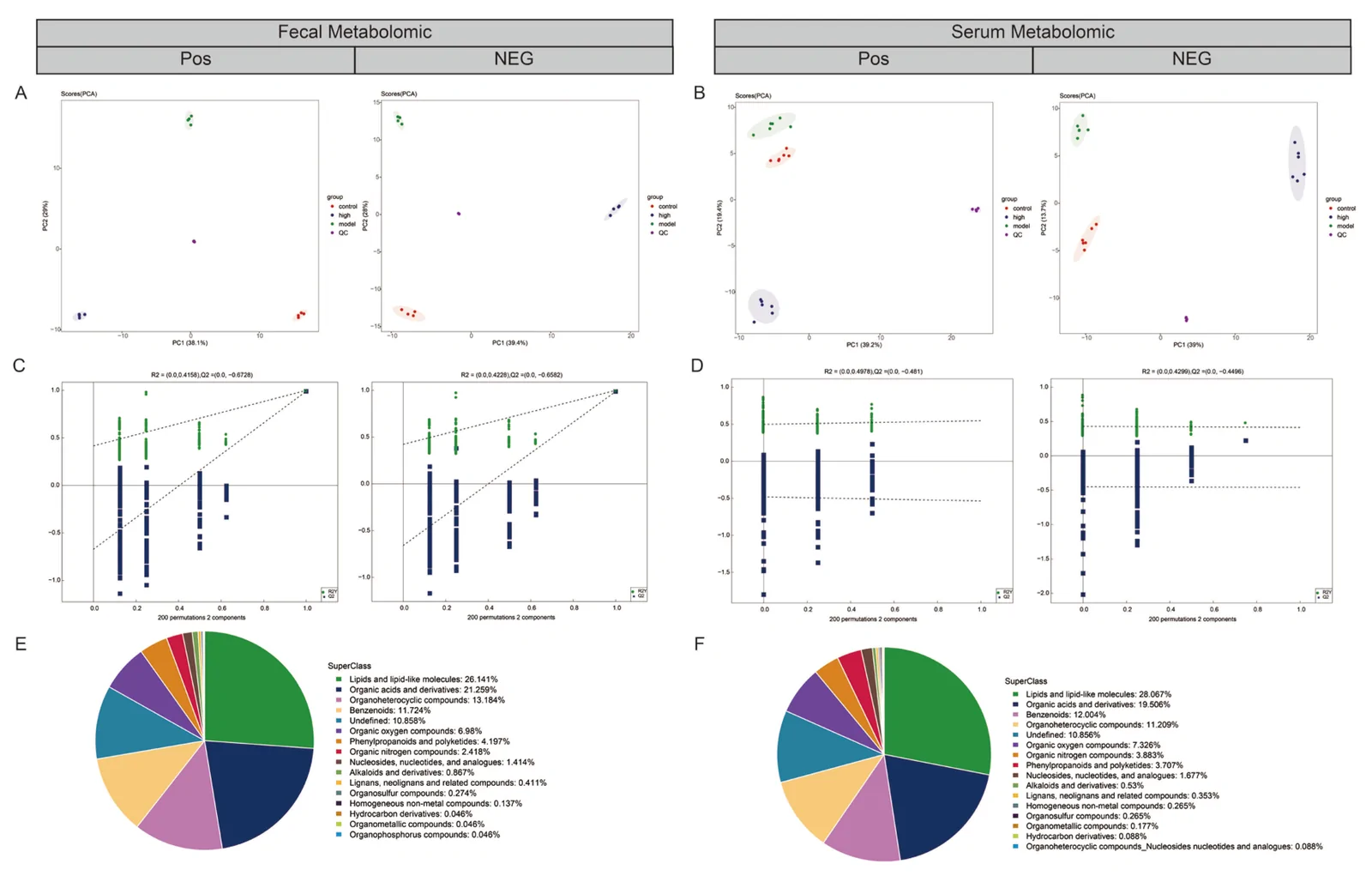
Fecal and serum metabolomic profiling of SQF-improved CIRI. (**A**,**B**) PCA score plots of fecal (**A**) and serum (**B**) metabolites in positive (Pos) and negative (Neg) ion modes, showing clear separation of control (red), SQF (dark blue), model (purple), and QC (green) groups and confirming data reproducibility and phenotypic differences. (**C**,**D**) Permutation test plots (200 permutations) for PLS-DA models of fecal (**C**) and serum (**D**) metabolites, with R2 and Q2 values approaching zero, validating model robustness and no overfitting. (**E**,**F**) Pie charts classifying identified fecal (**E**) and serum (**F**) metabolites by superclass, highlighting dominant classes such as lipids and lipid-like molecules, organic acids and derivatives, and organoheterocyclic compounds.

Regulating metabolism is one of the key pathways through which traditional Chinese medicine exerts its therapeutic effects [20]. Fecal and serum metabolomic profiling was performed in positive (Pos) and negative (Neg) ion modes. PCA score plots revealed clear separation among control, SQF, model, and quality control (QC) groups in both sample types, confirming analytical reproducibility and distinct metabolic phenotypes across experimental conditions (Figure 4A,B). Permutation tests validated the robustness of PLS-DA models, with R2 and Q2 values approaching zero, indicating no overfitting (Figure 4C,D). Classification of identified metabolites showed that lipids and lipid-like molecules, organic acids and derivatives, and organoheterocyclic compounds were the most abundant superclasses in both fecal and serum samples (Figure 4E,F).

**Figure 5:**
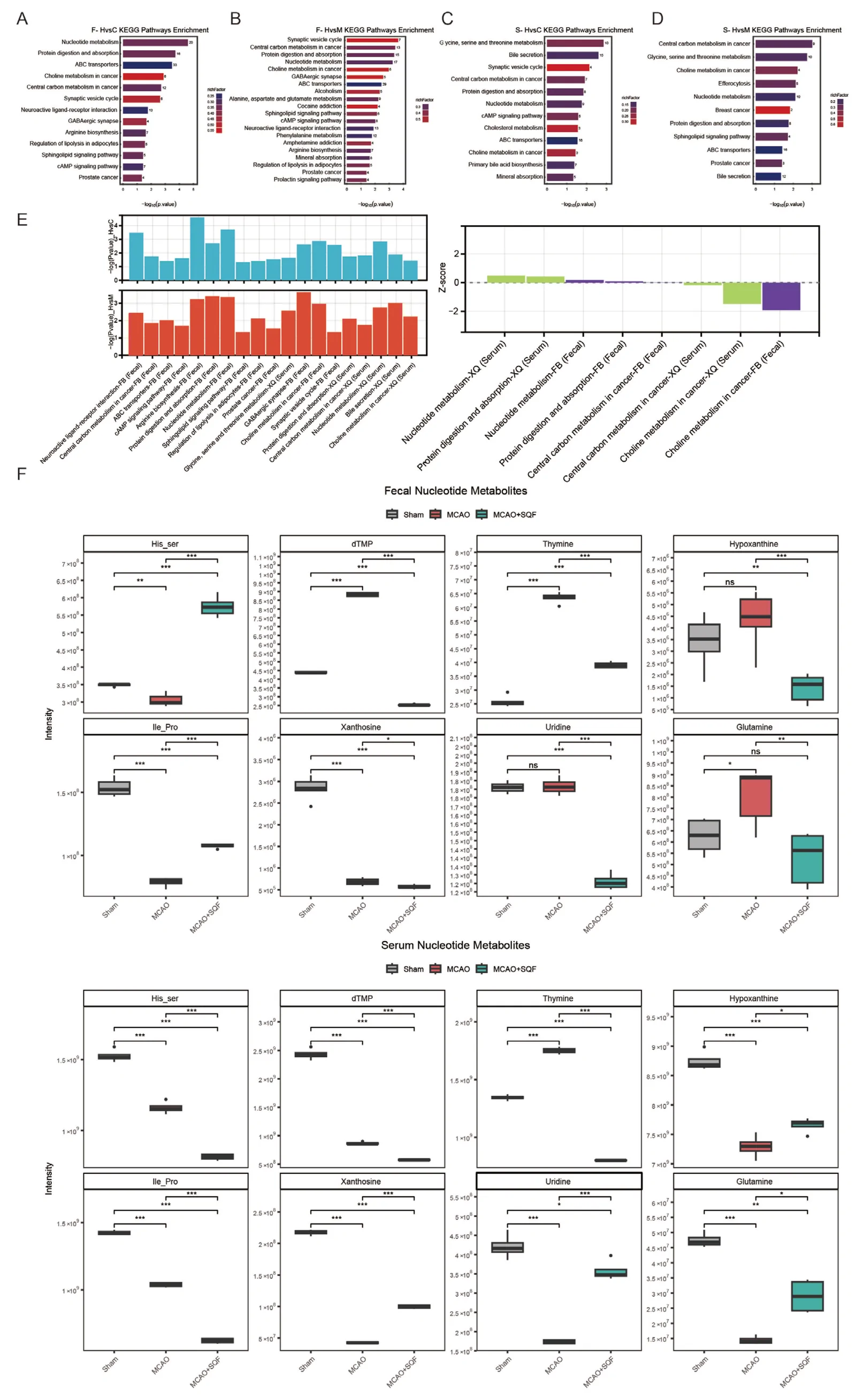
SQF improves CIRI by regulating nucleotide metabolism. (**A**–**D**) Bar charts of top enriched KEGG pathways for differential metabolites from fecal (**A**,**B**) and serum (**C**,**D**) samples. *X*-axis, −log_10_(*p*-value). Dot color indicates the rich factor. (**E**) Left, significance bar plot (−log_10_(*p*-value)) of shared enriched pathways between fecal and serum metabolome; right, Z-score plot showing overall up-/downregulation tendency of enriched pathways. (**F**) Box plots of key differential nucleotide-related metabolites in feces and serum across three groups (Sham, MCAO, MCAO + SQF). *Y*-axis represents metabolite signal intensity. * *p* < 0.05, ** *p* < 0.01, *** *p* < 0.001, ns, no significance.

To explore the modulatory effects of SQF on metabolic profiles in MCAO-induced cerebral-ischemia rats, untargeted metabolomics was performed on fecal and serum samples. KEGG pathway enrichment analysis revealed partially overlapping enriched pathways of differential metabolites between fecal (F-H vs. C, F-H vs. M, Figure 5A,B) and serum groups (S-H vs. C, S-H vs. M, Figure 5C,D), including nucleotide metabolism, protein digestion and absorption, ABC transporters, central carbon metabolism, sphingolipid signaling pathway and cAMP signaling pathway. Subsequent significance and Z-score trend analysis of shared pathways demonstrated remarkable disturbances in nucleotide metabolism, choline metabolism and central carbon metabolism (Figure 5E). The overall activity of these pathways was downregulated after MCAO surgery, and this abnormal trend could be reversed by SQF treatment (Table 2). Eight hub metabolites related to nucleotide and amino acid metabolism were selected for further validation. Box plots from fecal and serum samples showed that compared with the Sham group, the levels of His_ser, dTMP, Thymine and Xanthosine were significantly increased, while Ile_Pro was markedly decreased in feces of MCAO rats (Figure 5F).

**Figure 6:**
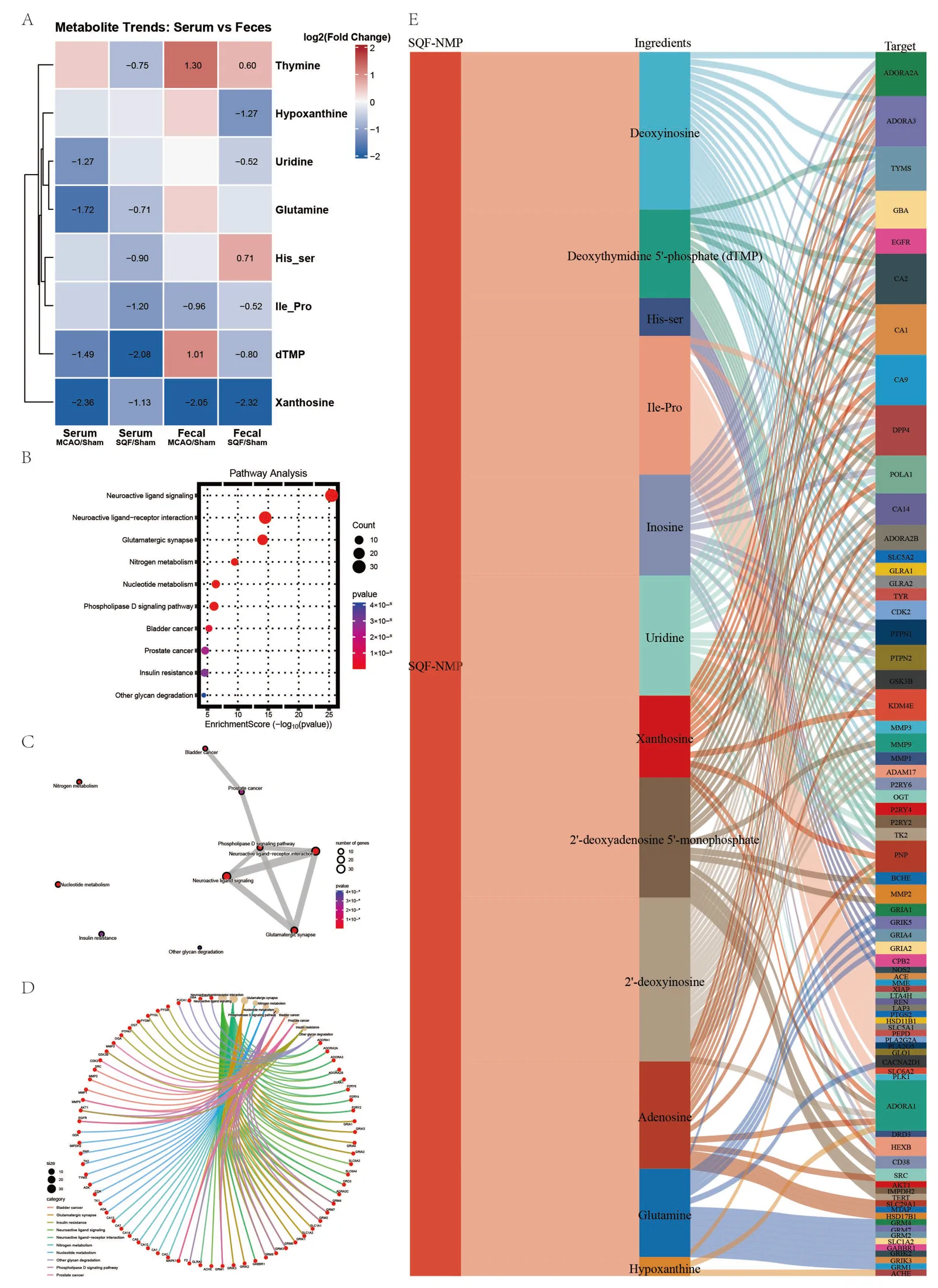
SQF improves CIRI by regulating nucleotide metabolism. (**A**) Heatmap displaying log_2_(fold-change) profiles of hub metabolites in serum and fecal samples. Red and blue colors denote increased and decreased metabolite abundance, respectively. (**B**) Bubble plot for KEGG enrichment analysis of altered metabolites. *X*-axis: enrichment score (−log_10_(*p*-value)). Dot size corresponds to metabolite count enriched in each pathway; dot color represents significance (*p*-value). (**C**) Metabolite–pathway interaction network. Node size indicates metabolite count; node color represents enrichment significance. (**D**) Chord diagram visualizing correlations between differential metabolites and enriched KEGG pathways. (**E**) Multi-level Sankey interaction network illustrating the “differential metabolite–ingredient–target” regulatory axis of SQF.

SQF intervention restored these metabolite levels. Serum metabolites exhibited a partly consistent changing tendency with fecal counterparts, indicating that cerebral ischemia triggered gut-serum nucleotide metabolic disorder, which could be rescued by SQF. The log_2_(fold-change) heatmap visualized the variation magnitude of core metabolites across groups in serum and feces (Figure 6A). Bubble enrichment analysis indicated that altered metabolites were mainly enriched in neuroactive ligand–receptor interaction, glutamatergic synapse, nucleotide metabolism and phospholipase D signaling pathway (Figure 6B). A Metabolite–pathway interaction network and a chord diagram further illustrated complicated correlations between metabolites and enriched pathways (Figure 6C,D). Finally, a multi-level Sankey network of “differential metabolite–ingredient–target” was constructed, showing that uridine is a key metabolite for regulating nucleotide metabolism in SQF (Figure 6E). Collectively, these results revealed that SQF could improve CIRI by regulating nucleotide metabolism.

### 3.4. Gut Microbiota Changes in SQF-Improved CIRI Are Closely Associated with Nucleotide Metabolism

**Figure 7:**
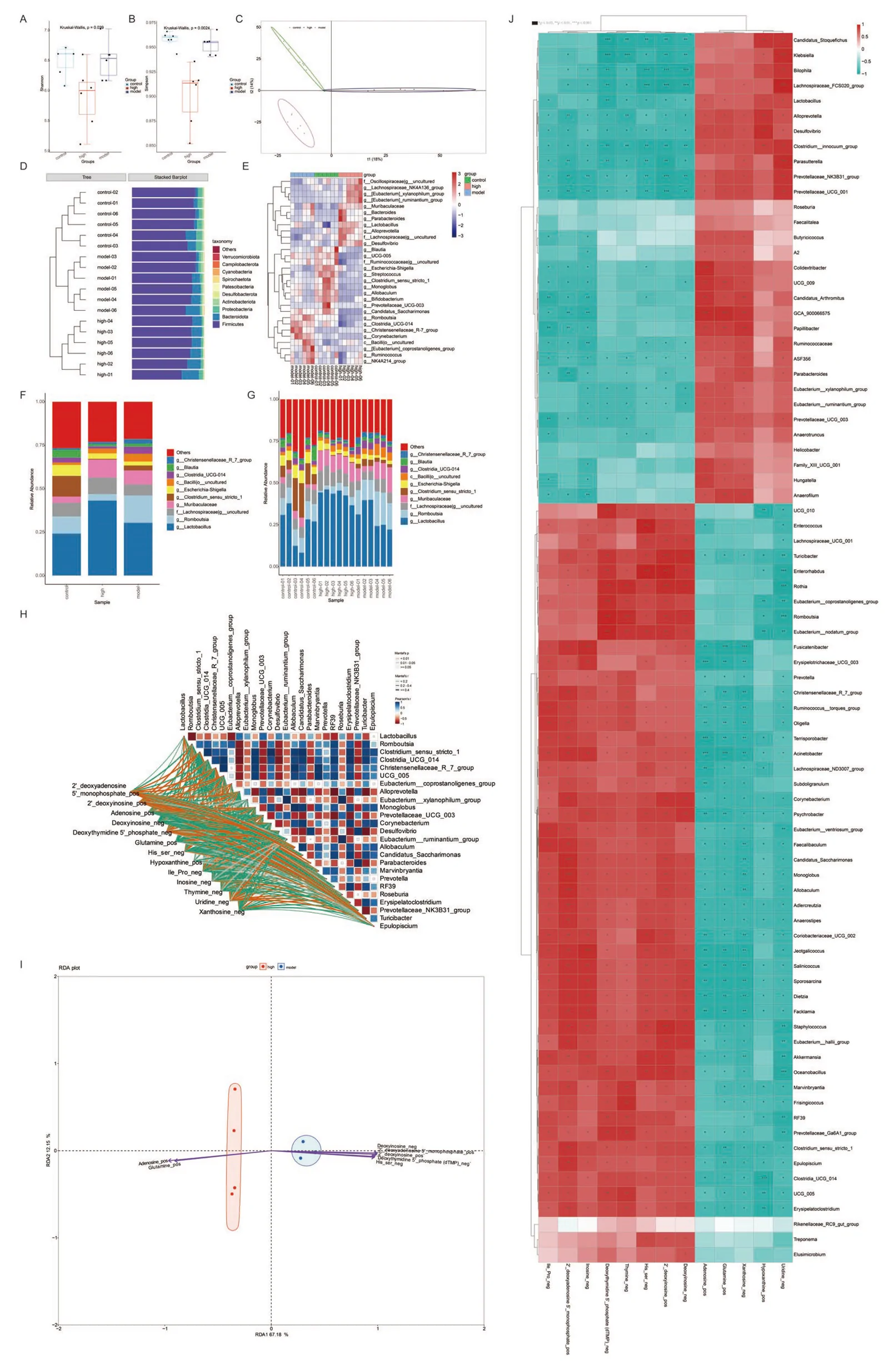
Gut microbiota changes in SQF-improved CIRI are closely associated with nucleotide metabolism. (**A**,**B**) Box plots of alpha diversity indices showing differences in microbial community richness and evenness among control, model, and SQF groups. (**C**) PCoA plot (beta diversity) illustrating distinct separation of microbial community structures between groups. (**D**) Hierarchical clustering dendrogram and stacked bar chart showing group-specific microbial patterns at the phylum level. (**E**) Hierarchical clustering heatmap of genus-level microbiota, showing group-specific microbial signatures. (**F**,**G**) Stacked bar charts displaying the relative abundance of gut microbiota at the phylum (**F**) and genus (**G**) levels. (**H**) Correlation heatmap between 13 key differential metabolites (involved in nucleotide metabolism) and top gut microbiota genera, colored by correlation strength. (**I**) RDA plot revealing the association between microbial communities, key metabolites, and experimental groups. (**J**) Correlation heatmap between 13 key nucleotide metabolites and gut microbiota (genus level), with hierarchical clustering to visualize positive and negative correlations.

The intestinal microbiota plays a crucial role in the prevention and treatment of various diseases [21], so we performed 16S rRNA gene sequencing analysis of fecal samples. Gut microbiota profiling revealed significant differences in community structure among control, model, and SQF-treated groups. Alpha diversity analysis showed altered richness and evenness in the model group, which was partially restored by SQF treatment (Figure 7A,B). PCoA demonstrated distinct separation of microbial communities between groups, confirming phenotype-specific microbiota signatures (Figure 7C). Hierarchical clustering further validated group-specific microbiota patterns (Figure 7D). Hierarchical clustering of the genus-level heatmap further confirmed the distinct microbial signatures among the control, model, and high groups (Figure 7E). Taxonomic classification identified changes in relative abundance at phylum and genus levels, with SQF treatment reversing model-induced dysbiosis (Figure 7F,G). The correlation heatmap visualized the strength and direction of associations between 13 key differential metabolites (primarily involved in nucleotide metabolism, e.g., adenosine, inosine, hypoxanthine, uridine, thymine, and deoxythymidine 5′-phosphate) and the top gut microbiota genera (Figure 7H). The RDA plot revealed distinct separation between the SQF-treated (red) and model (blue) groups along RDA1 (67.18%), confirming that SQF treatment reshapes the gut microbiota–metabolite axis, driving a distinct metabolic profile from the model state (Figure 7I). Correlation analysis revealed strong associations between 13 key nucleotide metabolites and gut microbiota (Figure 7J). Thus, gut microbiota changes in SQF-improved CIRI are closely associated with nucleotide metabolism.

### 3.5. Target Prediction of Blood-Entering SQF Components for CIRI Amelioration via Nucleotide Metabolism

**Figure 8:**
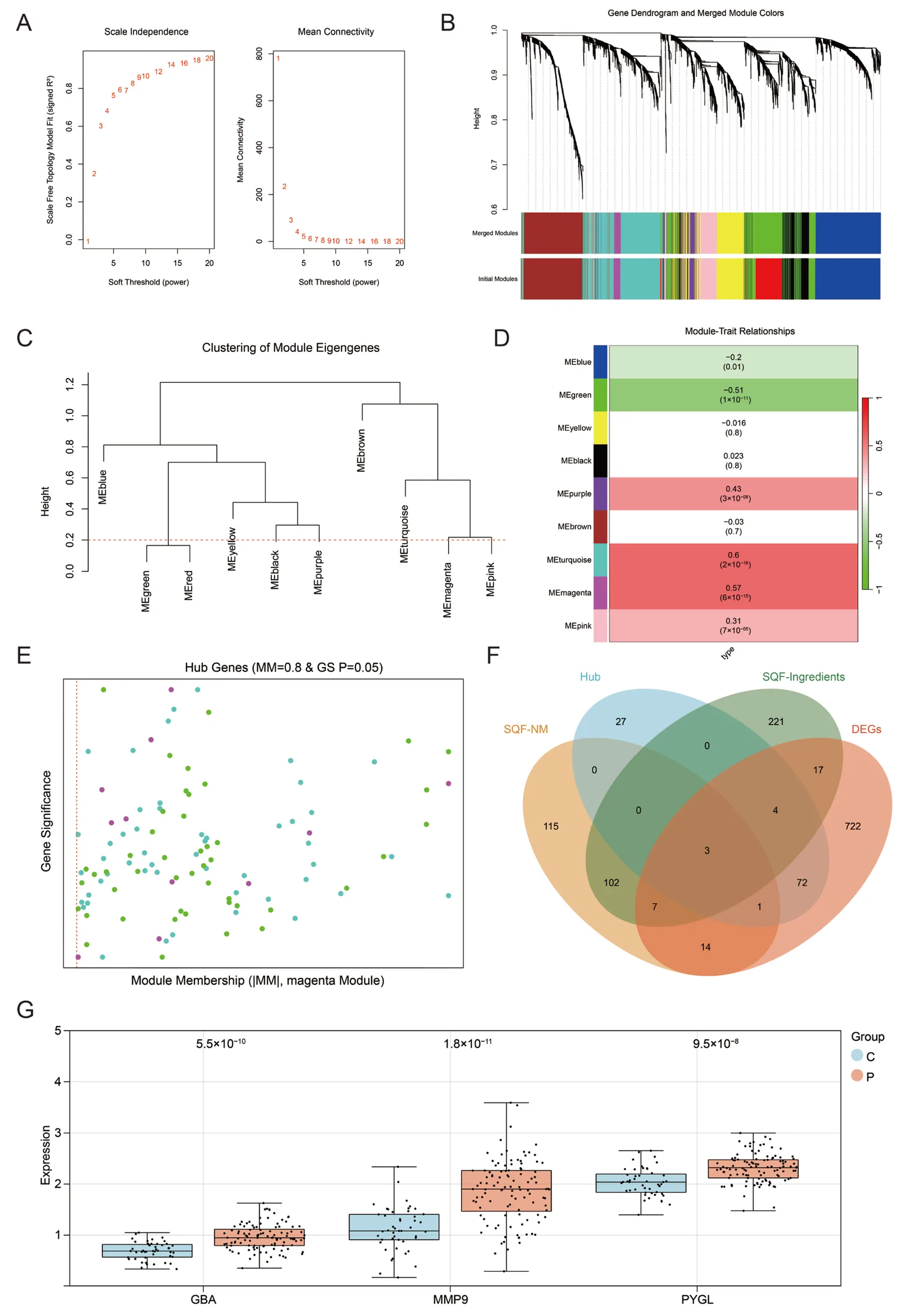
WGCNA and integrative analysis of SQF-related key genes. (**A**) Soft-thresholding power selection for WGCNA network construction, ensuring scale-free topology. (**B**) Gene dendrogram and co-expression module assignment (colored bars). (**C**) Clustering of module eigengenes. (**D**) Module–trait correlation heatmap. (**E**) Scatter plot for hub gene selection in the magenta module (MM > 0.8, GS *p* < 0.05). (**F**) Venn diagram showing overlap between hub genes, targets of SQF blood-entry ingredients, targets of SQF-nucleotide metabolites (SQF-NM), and DEGs. (**G**) Box plots validating dysregulation of hub genes (GBA, MMP9, PYGL) in control vs. patient samples.

WGCNA was performed to identify key modules and hub genes associated with IS. Soft-thresholding power selection (β = 12, Figure 8A) ensured scale-free topology, and hierarchical clustering (Figure 8B) partitioned genes into distinct co-expression modules. Module eigengene clustering revealed functional relatedness among modules (Figure 8C), and module–trait correlation analysis identified three key modules significantly associated with the IS phenotype: the turquoise module was strongly positively correlated (r = 0.60, *p* = 2 × 10^−16^), the magenta module was significantly positively correlated (r = 0.57, *p* = 6 × 10^−15^), and the green module was significantly negatively correlated (r = −0.51, *p* = 1 × 10^−11^) (Figure 8D). Hub gene selection in the magenta module identified genes with high module membership and gene significance (MM > 0.8, GS *p* < 0.05, Figure 8E). Venn diagram integration revealed three core overlapping genes (GBA, MMP9, and PYGL) among hub genes, SQF ingredient targets, and DEGs (Figure 8F). All three core genes passed one-to-one human–rat–mouse ortholog mapping; genes without stable one-to-one orthologs were excluded. Finally, box plot validation confirmed significant dysregulation of three hub genes (GBA, MMP9, and PYGL) between the control and model groups (Figure 8G), supporting their potential as key mediators of SQF’s protective effects.

### 3.6. Gba1 Gene Is the Key Target of SQF-Mediated CIRI Improvement

**Figure 9:**
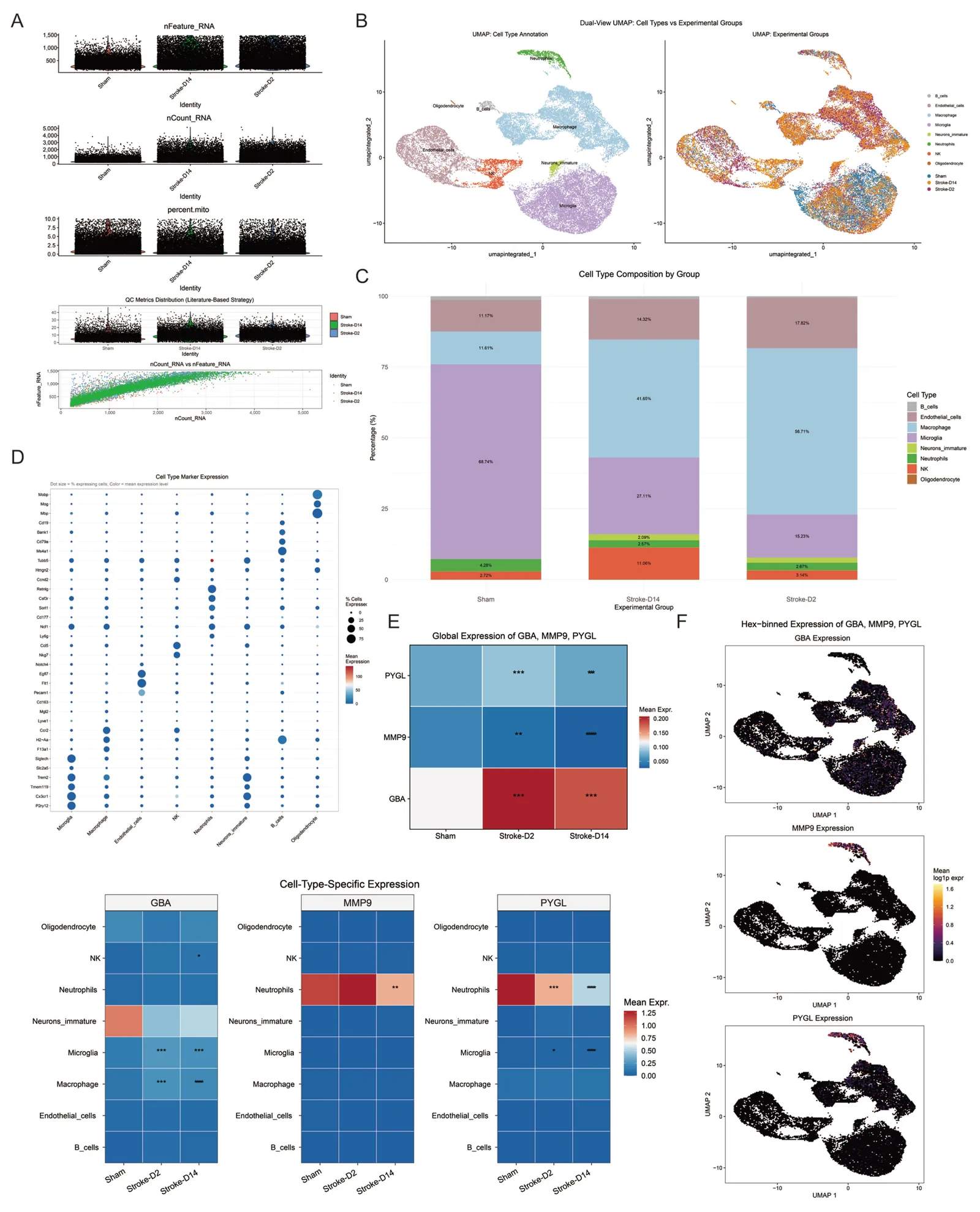
Cell-type-specific expression profiles of target genes *Gba1*, *Mmp9*, and *Pygl* in mouse brain after cerebral ischemia injury. (**A**) Violin plots for quality-control metrics of single-cell RNA-seq data. Distributions of nFeature_RNA, nCount_RNA, percent.mitochondrial genes and literature-based QC indicators are shown across three experimental groups (Sham, Stroke-D2: 2 days post-ischemia, Stroke-D14: 14 days post-ischemia). The scatter plot at the bottom illustrates the correlation between nCount_RNA and nFeature_RNA. (**B**) Dual-view UMAP plots. Cells are colored by annotated cell subtypes (left) and experimental groups (right). (**C**) Stacked-bar chart showing the proportional composition of different cell subtypes in brain tissues from Sham, Stroke-D2 and Stroke-D14 groups. (**D**) Dot plot of canonical cell-type marker genes. Dot size represents the fraction of cells expressing the marker; color intensity indicates the average gene-expression level. (**E**) Heatmaps of target-gene expression. Left: Global expression levels of GBA, MMP9 and PYGL across three groups. Right: Cell-type-specific expression heatmaps of the three target genes. * *p* < 0.05, ** *p* < 0.01, *** *p* < 0.001, ***** *p* < 0.0001. (**F**) Hex-binned UMAP plots visualizing the spatial distribution of GBA, MMP9 and PYGL across the whole cell atlas. Color gradient denotes mean log_10_-transformed expression values.

To further characterize the cellular landscape after cerebral ischemia and screen potential targets of SQF, single-cell RNA-sequencing was performed. Single-cell transcriptomic data of 12 brain tissue samples were successfully retrieved from the GEO database (GSE225948), including an initial total of 43,269 cells and 19,704 genes. After rigorous quality control filtering, a final total of 38,121 qualified cells were retained, with a cell retention rate of 88.1%. Visualization of quality control metrics showed uniform distribution of core indicators across the three groups without obvious abnormal peaks. The scatter plot exhibited a significant positive correlation between nCount_RNA and nFeature_RNA, indicating good cellular integrity and no severe RNA degradation (Figure 7A). The scDblFinder package was applied to detect and remove doublets. A total of 4444 doublets were identified, accounting for 11.7% of the initial valid cells. After doublet exclusion, 33,677 valid cells were retained for subsequent analysis (Figure 9A). Based on canonical marker gene sets, eight specific brain cell subtypes were successfully annotated, including microglia, macrophages, endothelial cells, NK cells, neutrophils, immature neurons, B cells, and oligodendrocytes (Figure 9B). The distribution profiles of the eight cell subtypes among the three groups are displayed in Figure 9C, revealing significant differences in cellular composition across groups. The marker gene expression heatmap demonstrated prominent cell-type specificity of core marker genes in each cell subtype (Figure 9D).

Three hub targets, GBA, MMP9 and PYGL, were selected for subsequent expression analysis. Global-expression heatmaps demonstrated that cerebral-ischemia injury significantly increased the transcript levels of these three genes compared with the Sham group. Cell-type-specific analysis revealed that GBA was predominantly enriched in microglia and macrophages, whereas MMP9 and PYGL were highly expressed in neutrophils (Figure 9E). Hex-binned UMAP plots further visualized the spatial high-expression regions of three hub target genes in ischemic lesions (Figure 9F).

**Figure 10:**
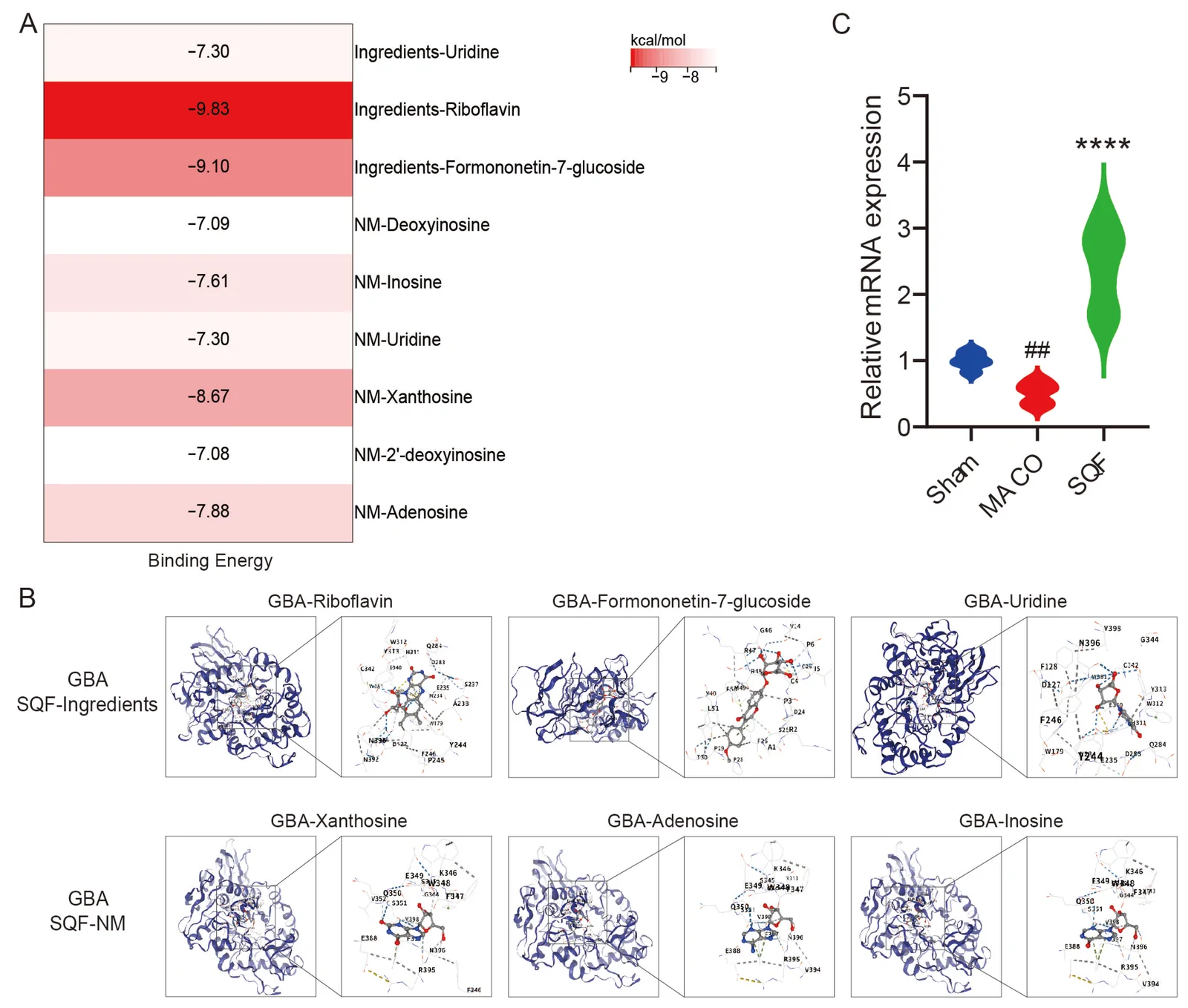
Molecular docking and expression validation of GBA with candidate compounds from SQF. (**A**) Heatmap showing the binding free energy of candidate ligands to GBA (unit: kcal/mol). Lower values indicate stronger ligand–protein binding affinity. (**B**) Three-dimensional molecular docking diagrams of GBA protein bound with SQF-Ingredients and SQF-NM (nucleotide metabolites). Enlarged panels display interacting amino acid residues and hydrogen-bond interactions within the binding pocket. (**C**) Relative mRNA expression of *Gba1* in rat brain tissues measured by RT-qPCR. ## *p* < 0.01 versus Sham group; **** *p* < 0.0001 versus MCAO group.

On the basis of multi-omics screening results, molecular-docking simulation was carried out between GBA protein and candidate compounds, including SQF-derived ingredients and nucleotide metabolites. Binding-energy data indicated that multiple compounds exhibited binding free-energy values lower than −7.0 kcal/mol. Riboflavin, formononetin-7-glucoside and xanthosine showed relatively strong binding affinity to GBA (Figure 10A). Three-dimensional docking diagrams showed that these ligands could stably fit into the active pocket of GBA and form hydrogen-bond interactions with key amino acid residues (Figure 10B). The GBA protein is encoded by the *Gba1* gene. RT-qPCR was then performed to verify the regulatory effect of SQF on *Gba1*. Compared with the Sham group, *Gba1* mRNA expression was significantly decreased in the MCAO group, and SQF treatment further significantly upregulated *Gba1* expression relative to the MCAO group (Figure 10C). Taken together, these findings indicate that SQF may exert protective effects against cerebral-ischemia injury via directly targeting and upregulating GBA.

### 3.7. Single-Cell Level Reveals the Cell-Specific Association Characteristics Between GBA and Uridine Metabolism

**Figure 11:**
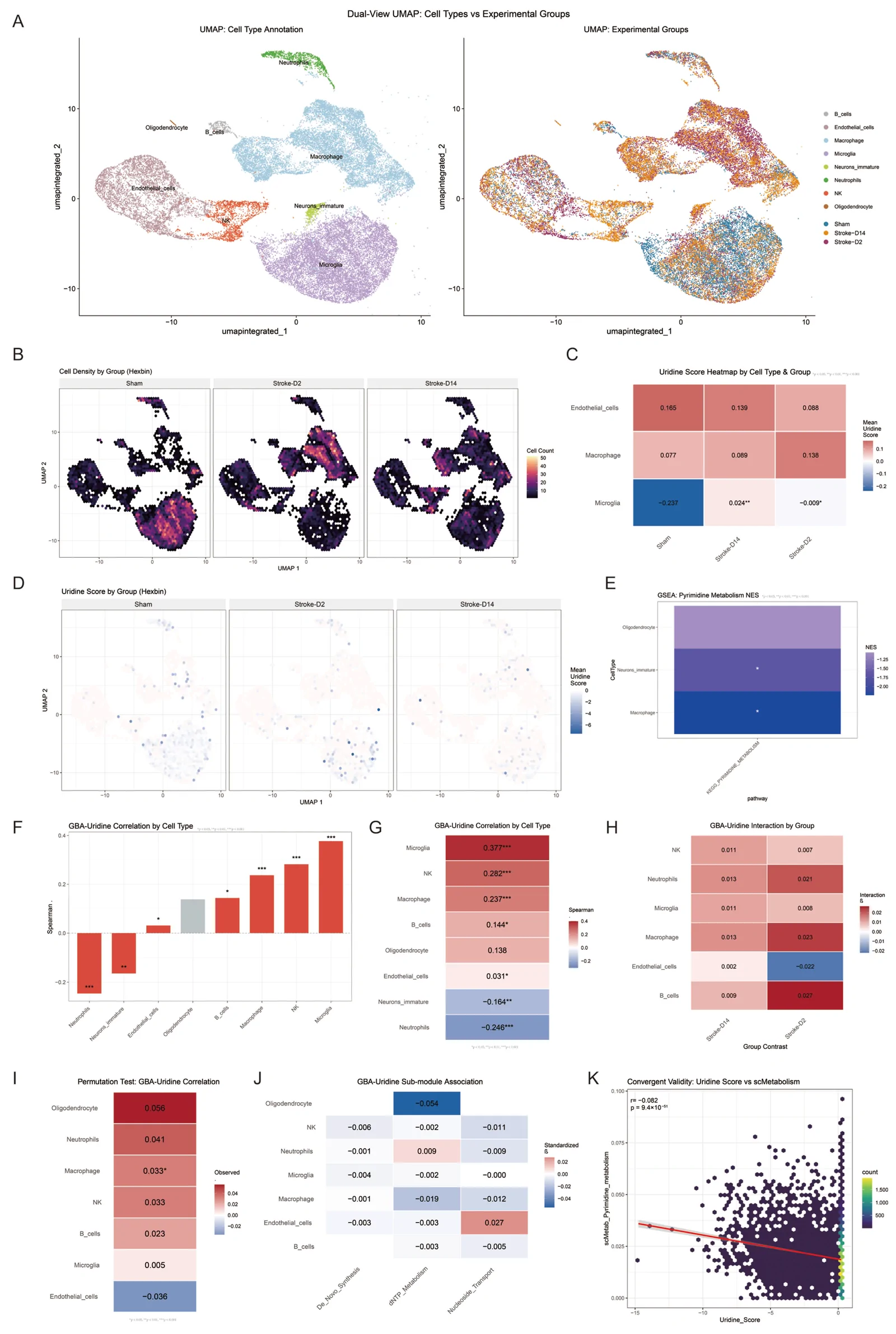
Cell-specific association between uridine–GBA and characteristics of pyrimidine metabolism at single-cell level after cerebral ischemia. (**A**) Dual-view UMAP plots. Cells are colored by eight annotated cell types (left) and three experimental groups (Sham, Stroke-D2, Stroke-D14) (right). (**B**) Hex-bin density UMAP plots visualizing spatial cell-density distribution across three groups. Color indicates cell count within each hex-bin. (**C**) Heatmap showing the mean uridine score of endothelial cells, macrophages and microglia in three groups. * *p* < 0.05, ** *p* < 0.01. (**D**) Hex-binned UMAP plots illustrating the spatial distribution of uridine scores across the cellular atlas in three experimental groups. Color gradient denotes mean uridine score. (**E**) Heatmap of normalized enrichment score (NES) for the pyrimidine-metabolism pathway (KEGG_PYRIMIDINE_METABOLISM) derived from GSEA in oligodendrocytes, immature neurons and macrophages. Asterisks indicate statistical significance. (**F**) Bar plot displaying Spearman’s correlation coefficients between GBA expression and uridine score across distinct cell types. * *p* < 0.05, ** *p* < 0.01, *** *p* < 0.001. (**G**) Heatmap showing Spearman correlation coefficients between GBA transcript level and uridine score in each cell subtype. Color intensity corresponds to correlation values. * *p* < 0.05, ** *p* < 0.01, *** *p* < 0.001. (**H**) Interaction-effect heatmap presenting standardized regression coefficient β values for the interaction between ischemic time (Stroke-D14, Stroke-D2) and the GBA–uridine association in individual cell populations. (**I**) Heatmap of permutation-test results for the GBA–uridine correlation within each cell type. Values represent observed effect sizes, * *p* < 0.05. (**J**) Heatmap of standardized regression β values showing associations between GBA–uridine correlation and three pyrimidine-metabolism sub-modules (De-Novo-Synthesis, dNTP-Metabolism, Nucleoside-Transport). (**K**) Hexagonal scatter plot for convergent-validity analysis, depicting the correlation between single-cell uridine score and single-cell pyrimidine-metabolic activity (scMetab_Pyrimidine_metabolism). The red line represents the linear-regression fit.

To explore the cell-specific association between uridine metabolism and hub gene GBA at single-cell resolution, uridine activity scoring, pathway enrichment and correlation analyses were performed. Dual-view UMAP plots illustrated the distribution of eight annotated cell subtypes and three experimental groups (Sham, Stroke-D2, Stroke-D14) across the cell atlas (Figure 11A). Hex-bin density maps revealed obvious spatial migration and redistribution of cells following ischemic injury (Figure 11B).

Single-cell metabolic scores were then compared among endothelial cells, macrophages and microglia at different ischemic time points. The uridine score was significantly increased in microglia at Stroke-D14, indicating an altered uridine transport/metabolism-associated transcriptional program in microglia in the late phase of cerebral ischemia (Figure 11C). Hex-binned UMAP visualization further exhibited spatial differences in uridine-score distribution across three groups (Figure 11D). GSEA results demonstrated divergent enrichment of the pyrimidine-metabolism pathway in oligodendrocytes, immature neurons and macrophages, suggesting ischemia-triggered reprogramming of pyrimidine metabolism in distinct cell populations (Figure 11E).

The Spearman correlation analysis between GBA expression and uridine score was calculated for each cell subtype. Significant positive correlations were observed in microglia, NK cells, macrophages, and B cells, while negative correlations were detected in neutrophils and immature neurons (Figure 11F,G). Importantly, these findings indicated a highly cell-specific GBA–uridine relationship, of which the correlation coefficient of microglia is the highest (Figure 11F,G). Interaction-effect analysis further revealed that ischemic duration modulated the strength of the GBA–uridine association in several cell types (Figure 11H). Permutation tests confirmed the statistical reliability of the correlations within macrophages and NK cells (Figure 11I).

We next investigated the links between the GBA–uridine association and three sub-modules of pyrimidine metabolism, including de novo synthesis, dNTP metabolism and nucleoside transport. Distinct contribution patterns of metabolic sub-modules were identified across different cell subtypes (Figure 11J). Convergent-validity testing showed a significant correlation between the single-cell uridine score and scMetab-derived pyrimidine-metabolism activity (r = −0.082, *p* = 9.4 × 10^−51^), verifying the reliability of our uridine-scoring metric (Figure 11K). Taken together, GBA is tightly coupled with the uridine transport/metabolism-associated transcriptional signature in a cell-specific manner under cerebral-ischemic conditions, with the strongest positive association found in microglia.

### 3.8. The Dynamic Evolution of GBA–Uridine Transport/Metabolism-Associated Transcriptional Coupling During Microglial Activation

**Figure 12:**
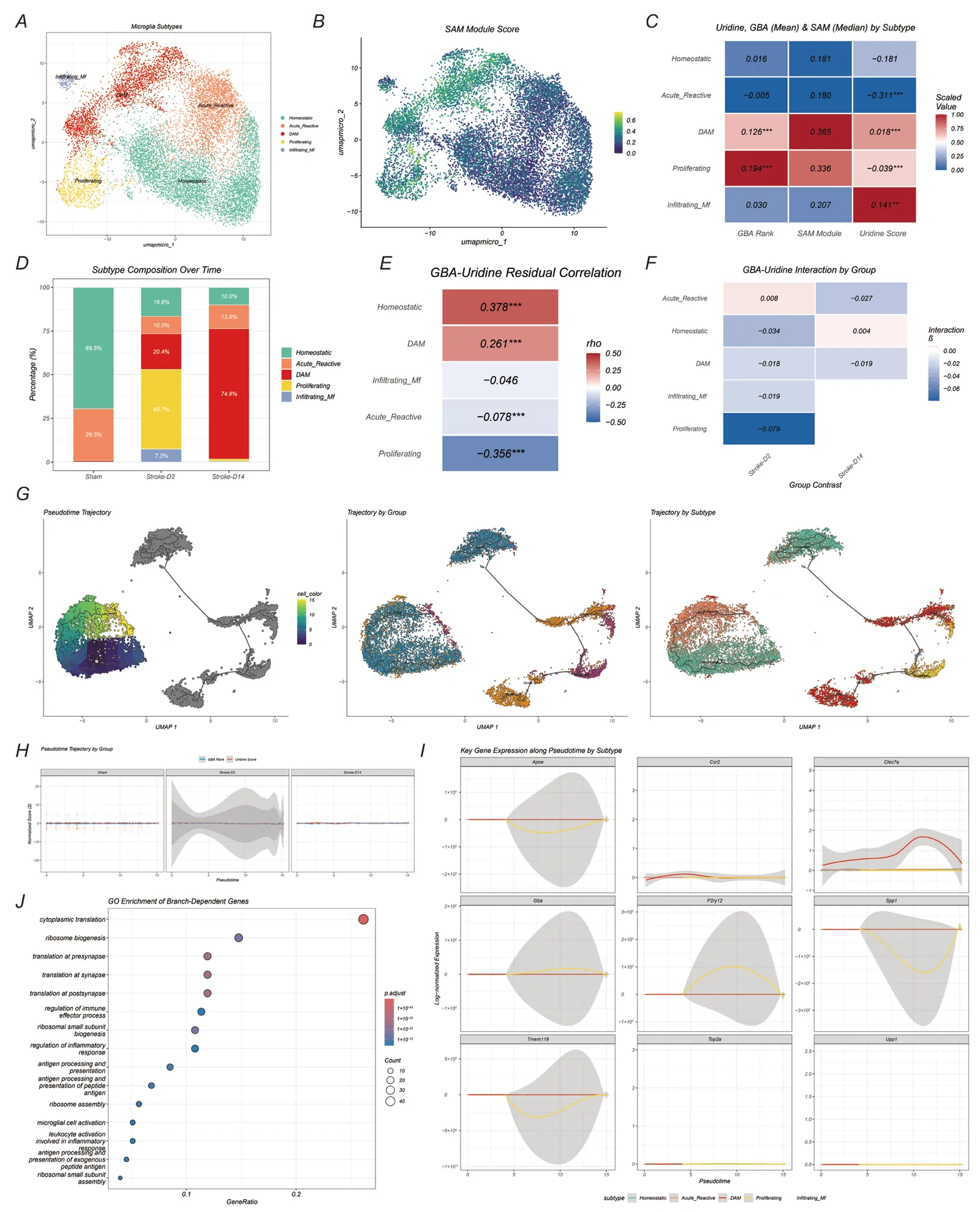
Sub-clustering, dynamic composition and pseudotime trajectory analysis of microglial populations. (**A**) UMAP plot of microglial subtypes. Five subpopulations were annotated, including homeostatic microglia, acute-reactive microglia, disease-associated microglia (DAM), proliferating microglia and infiltrating macrophages (Infiltrating_Mf). (**B**) UMAP plot colored by SAM module score. Color gradient indicates the activity level of SAM module. (**C**) Heatmap of scaled mean GBA rank value, median SAM-module score and mean uridine score across five myeloid subtypes. *** *p* < 0.001, ** *p* < 0.01. (**D**) Stacked-percentage bar chart illustrating dynamic proportions of microglial subtypes at three time points (Sham, Stroke-D2, Stroke-D14). (**E**) Heatmap of residual-adjusted Spearman’s correlation coefficients (rho) between GBA expression and uridine score within each microglial subtype. *** *p* < 0.001. (**F**) Interaction-effect heatmap showing standardized regression coefficient β values for the interaction between ischemic duration (Stroke-D2, Stroke-D14) and the GBA–uridine association across different subtypes. (**G**) Pseudotime trajectory plots for microglia. Left: Cells colored by pseudotime value; middle: Trajectory colored by experimental group; right: Trajectory colored by microglial subtype. Black lines indicate the main differentiation path. (**H**) Faceted trend plots depicting dynamic changes of GBA rank score and uridine score along pseudotime in Sham, Stroke-D2 and Stroke-D14 groups. Shaded areas represent 95% confidence intervals. (**I**) Pseudotime-dependent expression profiles of signature genes (*Apoe*, *Ccr2*, *Clec7a*, *Gba1*, *P2ry12*, *Spp1*, *Tmem119*, *Top2a*, *Upp1*). Log-normalized gene expression is plotted along the pseudotime axis, with colors corresponding to microglial subtypes. (**J**) Bubble plot of GO enrichment for branch-dependent differentially expressed genes from pseudotime analysis. *X*-axis represents GeneRatio, dot color denotes adjusted *p*-value (*p*.adjust), and dot size indicates the number of enriched genes.

Since our above-mentioned results demonstrated that microglia exhibited the strongest correlation between GBA and uridine metabolism, we next performed re-clustering on the microglial population to dissect the dynamic reprogramming of microglia after ischemic injury. Five microglia-related sub-populations were identified via unsupervised clustering: homeostatic microglia, acute-reactive microglia, disease-associated microglia (DAM), proliferating microglia and infiltrating macrophages (Infiltrating_Mf) (Figure 12A). SAM-module activity showed obvious regional enrichment on the UMAP map, indicating divergent activation status across these subtypes (Figure 12B).

Scaled values of GBA rank, SAM-module score and uridine score were then compared among subtypes. DAM and proliferating microglia displayed elevated GBA level, SAM activity and significantly increased uridine score, suggesting that these two activated populations serve as the major cellular compartments for the GBA–uridine axis (Figure 12C). Time-dependent compositional analysis revealed that homeostatic microglia dominated in the Sham group. At day 2 after ischemia, homeostatic microglia sharply decreased, while acute-reactive and proliferating subsets rapidly expanded. By day 14 post-stroke, DAM became the predominant microglial population, reflecting a progressive phenotypic shift from acute stress response towards a chronic disease-associated state (Figure 12D).

Residual-adjusted correlation analysis demonstrated a significant positive GBA–uridine correlation in homeostatic microglia and DAM, whereas remarkable negative correlations were observed in acute-reactive and proliferating subsets. This finding indicated that the polarity of the transcriptional coupling between GBA and the uridine transport/metabolism-associated signature reversed during distinct stages of microglial activation (Figure 12E). Interaction-effect analysis further suggested that ischemic duration exerted mild modulatory effects on GBA–uridine association strength within certain subtypes (Figure 12F).

Pseudotime trajectory reconstruction was applied to trace the full activation cascade of microglia. The trajectory delineated a continuous activation path originating from homeostatic microglia, progressing through an acute-reactive stage and finally differentiating into DAM. Group-colored trajectories confirmed that cerebral ischemia drove microglial cells to migrate along this activation route (Figure 12G). Pseudotime-dependent curves illustrated prominent fluctuations of GBA-rank score and uridine score at Stroke-D2 (Figure 12H). Pseudotime expression profiles of signature genes showed gradual downregulation of homeostatic markers (*P2ry12*, *Tmem119*) and progressive upregulation of activation-related genes (*Apoe*, *Spp1*, *Clec7a*, *Gba1*) along pseudotime (Figure 12I).

GO enrichment analysis of branch-dependent differentially expressed genes uncovered biological processes, including cytoplasmic translation, ribosome biogenesis, regulation of inflammatory response, microglial activation and antigen processing–presentation. These results implied robust translational reprogramming and inflammatory functional remodeling during DAM polarization (Figure 12J).

Taken together, cerebral ischemia triggers a continuous activation trajectory in microglia. GBA–uridine transport/metabolism-associated transcriptional association exhibits positive coupling in the homeostatic-to-DAM transition while presenting negative correlation during the early acute-proliferative phase. This dynamic switch may participate in orchestrating microglial polarization from resting status towards the pro-inflammatory DAM phenotype.

## 4. Discussion

This study integrated metabolomics, gut microbiota profiling, and single-cell RNA sequencing to elucidate the therapeutic mechanisms of SQF in a CIRI model. SQF reshaped the gut microbiota–metabolite axis and reversed nucleotide metabolic dysregulation. WGCNA and scRNA-seq suggested *Gba1* as a hub gene potentially linked to uridine metabolism, while molecular docking predicted high-affinity binding of SQF ingredients (e.g., riboflavin and formononetin-7-glucoside) and key nucleotide metabolites to GBA. Single-cell analysis further characterized the cell-specific GBA–uridine association and its potential role in microglial reprogramming after cerebral ischemia.

Many factors can lead to cerebral hemorrhage, possibly due to disruptions in the body’s metabolic products or pathways [22]. Therefore, traditional Chinese medicine can improve cerebral hemorrhage and reperfusion injuries by enhancing metabolism. UHPLC-Orbitrap MS identified 28 blood-absorbed components of SQF, including amino acids, nucleobases/nucleosides, an alkaloid, a vitamin, flavonoids, saponins, and organic acids. Most of these chemical classes are not unique to SQF, as many also occur in other medicinal herbs, foods, and traditional Chinese medicine formulas. Because SQF contains only Astragali Radix and Codonopsis Radix, its therapeutic specificity may rely on the combination, ratio, and multi-component/multi-target network of these two herbs rather than on a single unique compound. From a chemical standpoint, uridine (nucleoside core; pyrimidine metabolism), riboflavin (isoalloxazine ring; strongest GBA binding, −9.83 kcal/mol), and formononetin-7-glucoside (flavonoid glycoside; strong GBA binding) are key candidates. Xanthosine, inosine, adenosine, and related metabolites identified in metabolomic and docking analyses further link SQF to purine and pyrimidine metabolism. These nucleoside, flavonoid glycoside, isoalloxazine, and astragaloside/cycloastragenol saponin structures merit future structure–activity relationship studies. Whether these effects are specific to SQF or shared by other formulas containing Astragali Radix or Codonopsis Radix remains unclear. Comprehensive chemical isolation, component-knockout, and structure–activity relationship studies are beyond the scope of this work but will be important future directions.

The gut–brain axis acts as a critical bidirectional communication pathway between the intestinal tract and the central nervous system. Cumulative evidence indicates that gut microbiota dysbiosis disturbs host metabolic homeostasis and further aggravates neuroinflammation and brain damage after stroke [21,23]. Our 16S rRNA sequencing results confirmed obvious structural disorder of gut microbiota in MCAO model rats, while SQF treatment effectively restored the composition of gut flora. Combined with untargeted metabolomic analysis, we found that nucleotide metabolism was prominently disrupted in stroke rats, and significant correlations were observed between differential gut microbiota and altered nucleotide metabolites. These results demonstrated that SQF could remodel gut microbiota composition and correct abnormal nucleotide metabolism, thereby reversing the metabolic disturbance induced by cerebral ischemia.

Through WGCNA and scRNA-seq analysis, we screened three hub genes, *Gba1*, *Mmp9* and *Pygl*, that were closely linked to stroke pathology. Further cell-type localization revealed that *Gba1* was mainly expressed in microglia, macrophages and immature neurons closely related to IS, suggesting their potential roles in regulating neuroinflammation and neuronal function. Notably, this study revealed a cell-type-dependent coupling between GBA and uridine metabolism. The strongest positive correlation between GBA expression and uridine metabolic activity was observed in microglia (ρ = 0.377), whereas a negative correlation was detected in neutrophils. Such divergence may stem from distinct metabolic strategies adopted by different cell types. Upon transition toward the DAM phenotype after IS, microglia exhibit enhanced lysosomal activity. GBA-driven glycosphingolipid degradation may act in metabolic synergy with the uridine salvage-synthesis pathway to support cell-membrane remodeling and signal transduction. In contrast, neutrophils rely predominantly on glycolysis, and GBA function in these cells is likely linked to processes such as NETosis, leading to a fundamentally different mode of metabolic coupling. Loss-of-function studies from Gaucher- and Parkinson-related models have demonstrated that reduced GBA activity triggers intralysosomal accumulation of glucosylceramide, lysosomal stress and subsequent microglial over-activation [24]. Single-cell transcriptomic work has also reported robust enrichment of DAM-signature genes in GBA-deficient microglia, supporting a causal link between impaired GBA function and DAM transformation [25]. Notably, these previous findings are predominantly based on GBA-mutant or knockout systems, illustrating pathological outcomes induced by GBA insufficiency.

In contrast, few stroke-oriented single-cell studies have characterized dynamic wild-type GBA expression across sequential microglial activation stages (homeostatic-to-acute-reactive-to-proliferating-to-DAM). Metabolic crosstalk between GBA and pyrimidine–uridine metabolism in post-ischemic microglial subpopulations has not been documented. Our present study therefore provides novel complementary evidence from a wild-type ischemic-stroke model. The polarity-switching pattern of the GBA–uridine coupling we observed indicates that, beyond its well-known role in sphingolipid catabolism, GBA may cooperate with uridine-driven pyrimidine metabolism to orchestrate stage-dependent microglial phenotypic reprogramming following cerebral ischemia.

Little evidence indicates that GBA and nucleotide metabolism are closely interconnected, and that GBA does not act as a direct metabolic enzyme for nucleotides. As a lysosomal glucocerebrosidase, abnormal expression of GBA disrupts lysosomal function and subsequently impairs mitochondrial activity, which reduces the supply of ATP and key synthetic precursors required for de novo nucleotide synthesis, thereby disturbing the homeostasis of intracellular nucleotide pools [26,27,28]. In addition, GBA-mediated hydrolysis of glucocerebroside releases glucose, which participates in the turnover of sugar nucleotides such as UDP-glucose and further affects the balance of pyrimidine nucleotide metabolism [29,30,31]. More importantly, gut microbiota can modulate the transcription and activity of GBA via microbial metabolites, forming a regulatory loop linking gut flora, GBA and nucleotide metabolism [32,33,34]. Consistent with these mechanisms, our whole-tissue RT-qPCR results revealed that cerebral ischemia induced abnormal downregulation of *Gba1* (despite the microglial-specific upregulation observed in scRNA-seq) and disrupted nucleotide metabolism. Shenqi Granules could target GBA to restore lysosomal function, reverse metabolic reprogramming in microglia, and normalize uridine metabolism, which partially explains how this formula alleviates neuro injury through the gut microbiota–uridine–microglia axis.

The key advance of this study is not any single omics result but the fact that independent lines of evidence converge on the same axis. Blood-absorbed component profiling defined the pharmacological input of SQF and restricted subsequent analyses to compounds that actually reach the circulation. Network pharmacology and human IS transcriptomic WGCNA, although derived from different data sources and analytical frameworks, independently pointed to GBA as a shared core target. Serum and fecal metabolomics then showed that this target-level signal is accompanied by a functional metabolic shift, with uridine redistribution emerging as the dominant feature. 16S rRNA sequencing further indicated that this metabolic shift is not tissue-isolated: specific gut microbial taxa covaried with nucleotide metabolites, providing a plausible upstream driver of the circulating uridine changes. At single-cell resolution, the uridine transport/metabolism-associated signature was most strongly coupled with GBA in microglia and shifted dynamically along the homeostatic-to-DAM trajectory, thereby assigning the GBA–uridine relationship to a specific cell type and activation stage. Molecular docking and RT-qPCR connected the predicted ligand–GBA interactions with actual *Gba1* upregulation in the ischemic penumbra following SQF treatment. Collectively, these findings are mutually reinforcing rather than merely additive: the microbiota–metabolite correlation explains the source of uridine fluctuation, the single-cell data identify microglia as the responsive cell population, and the docking/RT-qPCR results link SQF components to GBA regulation. This convergence supports a working model in which SQF remodels the gut microbiota–uridine–GBA–microglia axis, and it also identifies specific nodes, particularly GBA and microglial uridine handling, that can be directly tested in future interventional studies.

Several limitations of this study should also be acknowledged. First, this study integrated human bulk transcriptomic data, rat in vivo experiments, and mouse single-cell RNA sequencing data. Although only one-to-one orthologs were retained for cross-species mapping, species-specific differences in gene regulation, immune responses, and metabolic networks may still affect the extrapolation of our findings. In particular, high-quality rat stroke scRNA-seq datasets with comparable time points remain limited, and the mouse dataset used here may not fully recapitulate the rat or human ischemic brain microenvironment. Second, we only verified the expression of hub genes at the transcriptional level; further protein-level detection and gene knockout/overexpression experiments are required to clarify the definite regulatory mechanism of *Gba1*. Third, the causal relationship between gut microbiota, uridine metabolism, and microglia activation needs more interventional evidence to confirm. Fourth, the pharmacodynamic material basis of SQF needs further exploration to clarify the exact effective components in vivo. However, it should be emphasized that Uridine_Score is a transcriptional metric derived from uridine transport- and metabolism-related genes. It reflects the coordinated transcriptional potential for uridine transport/turnover, rather than the actual intracellular uridine pool, uridine concentration, or metabolic flux [35]. Direct quantification of uridine and related metabolites (e.g., uridine, uracil, UDP, and UTP) in brain tissue, ideally combined with isotope tracing, will be required to validate the metabolic status inferred from this score [36]. In addition, batch-to-batch variation of SQF cannot be fully excluded, and establishing a standardized SQF reference sample with multi-batch fingerprint and quantitative marker analysis will be important for future reproducibility and clinical translation.

## 5. Conclusions

Collectively, SQF exerts its neuroprotective effects through modulating the gut microbiota–uridine metabolism–microglia activation axis, targeting hub genes like *Gba1* to regulate neuroinflammation and metabolic homeostasis. This study provides robust experimental evidence supporting the clinical application of SQF in ischemic stroke treatment and offers new insights into its multi-regulation therapeutic mechanism.

## Figures and Tables

**Table 1:** Identification results of blood-absorbed components of SQF. Blood-absorbed components were identified by UHPLC-Q-TOF-MS/MS in positive and negative ion modes. AR = Astragali Radix; CR = Codonopsis Radix; WD = Widespread; SQF-M = SQF-related metabolite. Source attribution is putative and does not imply exclusivity to SQF.

Number	Retention Time/min	Adduct Ion	[M+H]^+^/[M-H]^−^	Measured Value	Molecular Formula	Identified Compound	Compound Type	Source (Putative)
1	0.7	[M+H]^+^	175.118	175.1182	C_6_H_14_N_4_O_2_	Arginine	Amino acid	WD
2	0.8	[M+H]^+^	116.071	116.0711	C_5_H_9_NO_2_	Proline	Amino acid	WD
3	1.02	[M+H]^+^	136.062	136.0624	C_5_H_5_N_5_	Adenine	Nucleobase	WD
4	1.15	[M+H]^+^	130.05	130.0502	C_5_H_7_NO_3_	L-Pyroglutamic acid	Amino acid	WD
5	1.17	[MH]^−^	243.062	243.0629	C_9_H_12_N_2_O_6_	Uridine	Nucleoside	SQF-M
6	1.19	[M+H]^+^	182.081	182.0822	C_9_H_11_NO_3_	Tyrosine	Amino acid	WD
7	1.39	[M+H]^+^	132.102	132.1023	C_6_H_13_NO_2_	L-Isoleucine	Amino acid	WD
8	2.29	[M+H]^+^	166.086	166.0865	C_9_H_11_NO_2_	L-Phenylalanine	Amino acid	WD
9	2.8	[M+H]^+^	268.154	268.1538	C_14_H_21_NO_4_	Codonopsine	Alkaloids	CR
10	6.59	[M+H]^+^	377.146	377.1463	C_17_H_20_N_4_O_6_	Riboflavin	Vitamin	WD
11	7.77	[M+H]^+^	447.127	447.1277	C_22_H_22_O_10_	Calycosin-7-O-D-glucoside	Flavonoids	AR
12	11.04	[M-H]^−^	475.113	475.1224	C_22_H_22_O_9_	Formononetin-7-glucoside + HCOOH	Flavonoids	AR
13	12.42	[M+H]^+^	463.16	463.1599	C_23_H_26_O_10_	Methylnissolin-3-O-glucoside	Flavonoids	AR
14	13.13	[M-H]^−^	463.16	463.1599	C_23_H_28_O_10_	7,2′-dihydroxy-3′,4′-dimethoxyisoflavane-7-O-glucoside	Flavonoids	AR
15	13.15	[M-H]^−^	283.055	283.0597	C_16_H_12_O_5_	Acacetin	Flavonoids	WD
16	13.17	[M+H]^+^	285.076	285.0754	C_16_H_12_O_5_	Calycosin	Flavonoids	AR
17	21.68	[M-H]^−^	829.455	829.4544	C_41_H_68_O_14_	Astragaloside + HCOOH	Saponins	AR
18	21.69	[M+H]^+^	785.468	785.4676	C_41_H_68_O_14_	Astragaloside III	Saponins	AR
19	21.69	[M+H]^+^/[M+NH_4_]^+^	785.467	785.4676	C_41_H_68_O_14_	Astragaloside IV	Saponins	AR
20	23.65	[M+H]^+^	827.479	827.4783	C_43_H_70_O_15_	Isoastragaloside II	Saponins	AR
21	23.65	[M+H]^+^	175.06	175.0608	C_7_H_10_O_5_	Shikimic acid	Organic acids	WD
22	23.65	[M-H]^−^	871.465	871.4643	C_43_H_70_O_15_	Astragaloside II + HCOOH	Saponins	AR
23	23.67	[M+H]^+^	653.426	653.4266	C_36_H_60_O_10_	Cycloastragenol-6-O-β-D-glucoside	Saponins	AR
24	23.9	[M+H]^+^	943.526	943.5257	C_48_H_78_O_18_	Soyasaponin I	Saponins	AR
25	27.41	[M+H]^+^	869.489	869.4894	C_45_H_72_O_16_	Isoastragaloside I	Saponins	AR
26	27.41	[M-H]^−^	913.476	913.4755	C_45_H_72_O_16_	Astragaloside I + HCOOH	Saponins	AR
27	30.6	[M+H]^+^	911.5	911.5006	C_47_H_74_O_17_	Acetylastragaloside I	Saponins	AR
28	35.94	[M+H]^+^	297.242	297.2413	C_18_H_32_O_3_	9(S)-HODE	Organic acids	WD

**Table 2:** RM and Z-score characteristics of KEGG pathways in fecal and serum metabolomics.

Map_ID	Pathway Name	Sample Type	−log (*p*-Value)_H vs. M	−log (*p*-Value)_HvsC	Z-Score
rno01232	Nucleotide-metabolism	FB-(Fecal)	3.24	4.61	0.186
rno05230	Central-carbon-metabolism-in-cancer	FB-(Fecal)	3.41	2.71	0.009
rno04974	Protein-digestion-and-absorption	FB-(Fecal)	3.36	3.72	0.084
rno05231	Choline-metabolism-in-cancer	FB-(Fecal)	2.97	2.88	−1.923
rno01232	Nucleotide-metabolism	XQ-(Serum)	2.11	1.75	0.497
rno04974	Protein-digestion-and-absorption	XQ-(Serum)	1.75	1.82	0.419
rno05230	Central-carbon-metabolism-in-cancer	XQ-(Serum)	3.01	1.9	−0.173
rno05231	Choline-metabolism-in-cancer	XQ-(Serum)	2.23	1.44	−1.487

## Data Availability

The original contributions presented in this study were included in the article. Further inquiries can be directed to the corresponding authors.

## Abbreviations

ANOVA, One-way analysis of variance; ASVs, Amplicon sequence variants; BP, Biological process; CC, Cellular component; CCA, Common carotid artery; CIRI, Cerebral ischemia-reperfusion injury; DAM, Disease-associated microglia; DEGs, Differentially expressed genes; DL, Drug-likeness; ECA, External carotid artery; GBA, glucocerebrosidase (GCase); GO, Gene Ontology; GS, Gene significance; HRP, Horseradish peroxidase; ICA, Internal carotid artery; KEGG, Kyoto Encyclopedia of Genes and Genomes; KO, Knockout; MAD, Median absolute deviation; LR, Ligand-receptor; MCAO, Middle cerebral artery occlusion; MF, Molecular function; MM, Module membership; MWM, Morris water maze; NOR, Novel object recognition; OB, Oral bioavailability; OFT, Open field test; OTUs, Operational taxonomic units; PCA, Principal component analysis; PCoA, Principal co-ordinate analysis; PLS-DA, Partial least squares discriminant analysis; Q2, Predictive ability; QC, Quality control; R2, Goodness of fit; RDA, Redundancy analysis; RT-qPCR, Reverse transcription–quantitative real-time PCR; scRNA-seq, Single-cell RNA sequencing; SD, Standard deviation; SMILES, Simplified molecular input line entry specification; SQF, Shenqi granules; TCM, Traditional Chinese medicine; TCMSP, Traditional Chinese Medicine Systems Pharmacology; TOM, Topological Overlap Matrix; TTC, 2,3,5-Triphenyltetrazolium chloride; UMAP, Uniform manifold approximation and projection; WGCNA, Weighted gene co-expression network analysis.
